# Modeling and simulation analysis of multi-scenario air conditioning cluster response for intelligent load management in distribution networks

**DOI:** 10.1038/s41598-025-99816-w

**Published:** 2025-05-14

**Authors:** ZhiYong Zhang, HaiYun Wang

**Affiliations:** Engineering Research Center of Education Ministry for Renewable Energy Power Generation and Grid Connection, Xinjiang University, Urumqi, 830017 China

**Keywords:** Human Activity Modeling, Intelligent Load Management, Dynamic Scenarios, Air Conditioning Load Response, Cluster Response Modeling, Electrical and electronic engineering, Renewable energy

## Abstract

This paper proposes a dynamic multi-scenario modeling approach for air conditioning (AC) cluster loads, integrating occupant behavior, spatiotemporal activity distributions, and meteorological factors. A refined unregulated load baseline is established to better isolate and evaluate the effects of AC usage on overall distribution network loads. Simulation results under various scenarios indicate that the proposed framework accurately captures cluster-level load responses, effectively reflecting the interplay among occupant activities, temperature variations, and regional characteristics. The outcomes demonstrate the model’s potential to enhance AC load forecasting and support intelligent demand-side management in smart grids, offering both theoretical and practical insights for future load regulation strategies.

## Introduction

As global warming becomes an increasingly urgent issue, major economies are facing unprecedented pressure to reduce energy consumption and carbon emissions^1^. According to the goals outlined in the Paris Agreement, substantial reductions in greenhouse gas emissions are required globally, which presents an urgent demand for the transformation of energy structures. In recent years, traditional fossil fuels have been gradually restricted, while clean energy sources such as wind and solar power have attracted significant investments due to their environmental advantages^2^. However, the volatility and intermittency of these clean energy sources limit their stable replacement of conventional thermal power plants, posing challenges to grid stability^3^.

In response to these challenges, countries have been actively developing emerging technologies such as energy storage, virtual power plants, and demand response, all aimed at enhancing the absorption capacity of clean energy and improving the stability of power systems^4^. Among these, demand response technology has become a research hotspot due to its ability to achieve precise load regulation at the consumer end^5^. However, the effectiveness of demand response is highly dependent on user electricity consumption behavior, particularly the impact of complex and variable air conditioning loads on regional supply-demand balance. Air conditioning, as a typical high-energy-consumption load, is influenced by multiple factors such as environmental temperature, user demand, and equipment characteristics^6^. Studies have shown that in summer, air conditioning loads contribute to over 40% of the grid’s peak demand, making their regulation a critical element in peak shaving, valley filling, and load optimization. For instance^7^, demonstrated that smart electricity technologies, through demand response mechanisms, enable flexible interaction between the grid and users, significantly enhancing the regulation capability of air conditioning loads. Additionally^8^, proposed an adaptive PID control method for photovoltaic air conditioning systems, which effectively improves power tracking performance while maintaining indoor thermal comfort^9^. further validated the potential of air conditioning loads in energy savings and thermal comfort through an optimized control strategy for multi-zone variable air volume systems.

However, the response behavior of air conditioning loads at the cluster level exhibits significant diversity and randomness, presenting substantial challenges for modeling. For example^10^, developed a cluster air conditioning load model based on heat exchange balance principles and verified the feasibility of air conditioning loads participating in microgrid frequency regulation by adjusting target temperatures^11^. proposed an emergency demand response strategy based on the whale optimization algorithm, which effectively improves power system voltage stability while reducing fluctuations in air conditioning loads^12^. integrated active and passive heating, cooling, lighting, shading, and ventilation systems, significantly reducing building energy consumption and improving user comfort.

Most existing research on air conditioning load modeling focuses on single scenarios or specific regions. While these studies provide valuable insights into the characteristics of air conditioning load response, they often fail to fully capture the impact of multi-scenario and multi-factor interactions on collective load response characteristics. For instance^13^, proposed an aggregate flexibility model for thermostatically controlled loads, treating air conditioning loads as a stochastic battery model to effectively capture their dynamic response characteristics^14^. introduced a control strategy for aggregated air conditioning loads based on room thermal models, significantly improving reserve capacity and response time by resetting temperature setpoints^15^. modeled air conditioning loads as virtual energy storage devices, demonstrating their potential in providing regulation services^16^. implemented a cloud-based platform for smart building interconnection and proposed a distributed load control algorithm based on comfort levels and charging priorities, further expanding the application of air conditioning loads in demand response.

Existing air conditioning load modeling methods can be broadly classified into the following categories:

1. **Physics-based methods**: These methods simulate air conditioning load response behaviors using heat balance equations. For example, in^17^, a model based on the indoor-outdoor temperature differential is proposed, which can accurately simulate the start-up and shutdown states of the equipment. These approaches provide a solid theoretical foundation for air conditioning load modeling but rely heavily on numerous physical parameters (e.g., room thermal capacitance and heat dissipation coefficients), making parameter acquisition challenging in complex environments^18,19^. Within this category, multi-scenario modeling extends the framework by classifying loads across residential, commercial, and industrial contexts, integrating scenario-specific variations (e.g., regional climate or building types) with heat balance principles. However, such models often focus on static analysis, neglecting dynamic factors like inter-regional human movement and time-varying interactions that influence load characteristics^20,21^.
2. **Data-driven methods**: These methods train models using historical data and employ machine learning techniques to predict air conditioning load profiles. They offer computational efficiency and can quickly fit existing data, but often fail to capture dynamic response characteristics, limiting adaptability in real-time demand response scenarios^20–22^. In some cases, data-driven techniques complement physics-based approaches, including multi-scenario modeling, by providing empirical trends to refine predictions.

Although the aforementioned studies provide valuable theoretical insights for air conditioning load modeling, most methods struggle to effectively capture the diversity and dynamic characteristics of air conditioning cluster load responses in complex environments and diverse scenarios^23^. The regulation behavior of air conditioning loads is not only influenced by ambient temperature and user demand but also closely related to residents’ time-use behavior^24^. Therefore, accurately modeling the dynamic response characteristics of air conditioning loads, especially the cluster response behavior in multiple scenarios, has become a current research hotspot and challenge. To address this, the present study proposes a novel approach, wherein the activity states of individuals in multiple scenarios are simulated as the driving force for air conditioning cluster behavior, thereby constructing a multi-scenario air conditioning cluster response model for intelligent distribution network load management. While intelligent load management increasingly incorporates advanced algorithms to enhance demand response capabilities^25–27^, this study prioritizes a physics-based approach to model air conditioning cluster responses across multiple scenarios. This framework aims to capture the dynamic influences of occupant behavior and environmental factors, providing a foundation for future integration with algorithmic techniques.

**Fig. 1:**
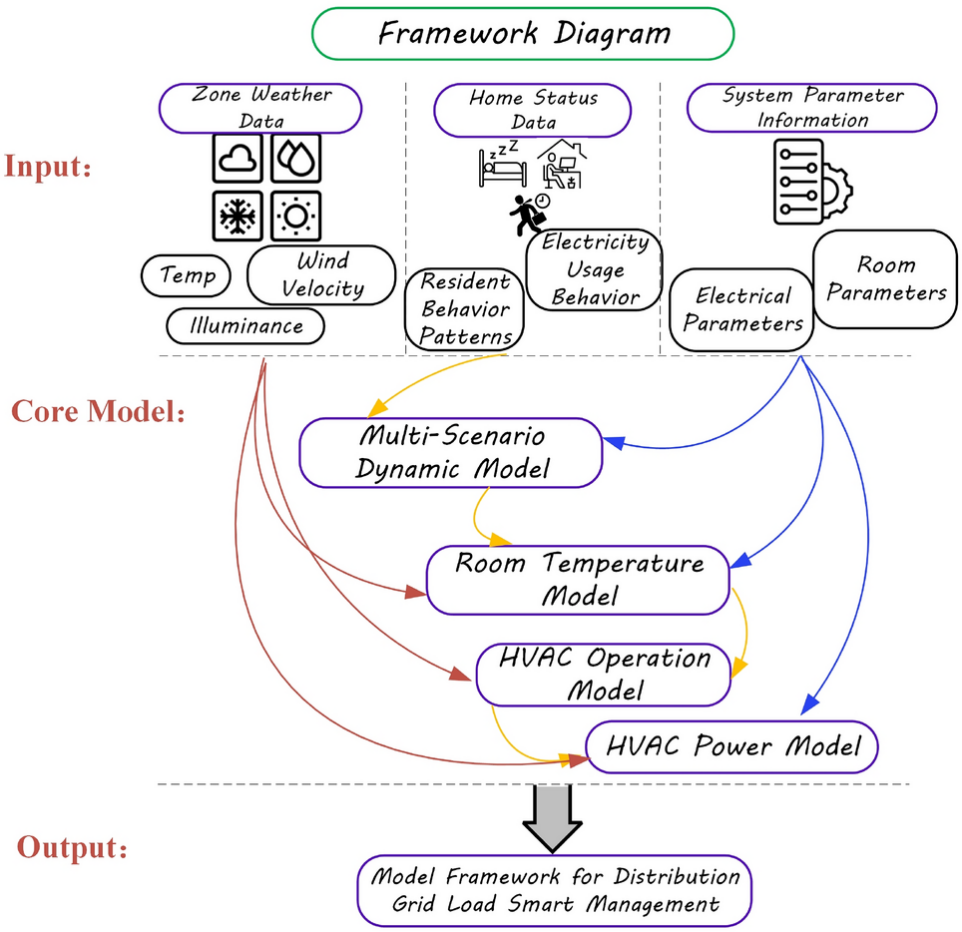
Model framework diagram.

**The main contributions of this study**: A multi-scenario dynamic model is developed, including a time-space dynamic distribution model for residential activities and a non-controllable load dynamic change model. Based on the principle of heat balance, meteorological data and location-specific characteristics are integrated to derive an indoor temperature model, and an air conditioning operation signal model is developed that aligns with the dynamic behavior of individuals. Furthermore, considering the temperature differential environment, an energy consumption model for the air conditioning cluster is established, quantifying the response patterns of air conditioning load across multiple scenarios. The model framework is illustrated in figure 1.

## Multi-scenario dynamic model setup

The multi-scenario dynamic model includes a time-space dynamic distribution model for residential activities and a distribution network non-controllable load model. This section provides a detailed description of the basic setup of the model, along with an explanation of the interrelationships and logical support.

### Time-space dynamic distribution setup for the distribution network

This part simulates the dynamic distribution characteristics of residential activities within the distribution network using both temporal and spatial dimensions:

- **Temporal Dimension**: Based on date types (e.g., weekdays, weekends, holidays) and different time periods within a day, the model simulates the patterns of residential activities and load variations. Load fluctuations are captured through time series mapping, reflecting changes in electricity demand during various periods of the day.
- **Spatial Dimension**: Considering commuting times between residential areas and various functional zones (such as administrative districts, industrial zones, and commercial districts), this aspect accounts for the impact of regional locations on population distribution. It then estimates the load response characteristics across different regions based on this spatial distribution.

### Dynamic activity setup for the distribution network population

- **Baseline Population for Regions**: The baseline population of residential areas is determined by the number of households and the proportion of family sizes, while the baseline population of functional areas is calculated based on the allocation coefficients between residential areas and various functional zones.
- **Activity States within Regions**: Within residential areas, individuals are classified into three activity states-resting, active, and away-based on their home status. In functional areas, it is assumed that individuals are always in an active state to reflect the specific load demand patterns of those regions.
- **Inter-Regional Population Migration**: The model assumes that only migration between residential areas and functional zones is considered, and migration between functional zones is neglected to simplify computational complexity. Migration data is used to analyze the dynamic characteristics of the population in areas such as commercial districts.

### Dynamic load variation rules for distribution network regions

This section describes the dynamic characteristics of each region based on the classification of regional types and load patterns.The simulation characteristics for different region types are summarized in Table 1.

**Table 1:** Simulation Characteristics by Region Type.

Region Type	Dynamic Characteristics	Load Pattern	Simulation Setup Description
Residential Area	Significant daily activity patterns	Strong consistency in electricity load composition	Simulates energy consumption of the resident population in different areas
Administrative Area	Fixed working hour patterns	Stable load	Simulates the work load in centralized office areas
Industrial Area	Mixed shift and non-shift operations	Strong regularity in high load	Simulates high-energy consumption industrial parks located away from urban centers
Commercial Area	Significant fluctuations in population flow	High volatility and uncertainty	Simulates the fluctuating characteristics of commercial activities

**Note**: This model allows for flexible adjustments of baseline populations and allocation coefficients to meet the specific economic and social characteristics of different regions.

The above setup forms the foundation for the construction of the multi-scenario dynamic model. Based on this foundation, the model is further divided into the following two main parts:

- **Time-Space Dynamic Distribution Model for Residential Activities**: Describes the dynamic changes in the population within the distribution network and simulates their impact on load.
- **Non-Regulated Load Dynamic Variation Model**: Simulates load variation patterns across different regions and captures the load fluctuation characteristics.

## Time-space dynamic distribution model for residential activities

The time-space dynamic distribution model of residential activities is crucial for understanding air conditioning load response behavior. Studies have shown that there is a significant causal relationship between residents’ time-use behavior and energy consumption^24^. For instance, residents’ activity states during different time periods (resting, active, or going out) directly influence the frequency of air conditioner usage and energy consumption^28^. Therefore, this paper constructs a time-space dynamic distribution model to simulate residents’ activity patterns in different scenarios, and integrates this with a non-regulated load model to quantify regional load changes.

### Dynamic modeling of residential area population

In the residential area, households are considered the basic unit, with family sizes ranging from 1 to 6 people, serving as a key factor in modeling occupant behavior for intelligent load control in urban settings. The distribution of family sizes varies by region, but for this study, a representative distribution is proposed based on general urban demographic trends observed in recent years^29,30^. As shown in Table 2, families of 1 to 3 members are assumed to dominate, with 2-member families comprising a significant portion, reflecting urban environments where energy demand is influenced by population density and lifestyles. These proportions are intentionally broad and hypothetical, designed to capture a range of plausible scenarios rather than a specific dataset. In the model, family size proportions are randomly generated within these ranges (summing to 100%) by default, with the flexibility to manually input specific proportions for tailored scenarios.

**Table 2:** Family Size Distribution in Urban Residential Areas.

Family Size	Proportion (%)
1	20%–35%
2	30%–40%
3	20%–30%
4	5%–15%
5	1%–5%
6	0%–2%

**Note:**Proportions are hypothetical and broad, inspired by urban trends^29,30^, with random generation and optional manual input.

### Home State Matrix (HSM)

#### Description of the state matrix

The home behavior states of residents in the residential area vary with time periods and are mainly divided into the following three states:

- **Resting State**: Residents are in a sleeping state.
- **Active State**: Residents are engaged in household chores, entertainment, etc., at home.
- **Out State**: Residents are either out or working.

Thus, the home status matrix (HSM) can be represented as:

$$HSM = \begin{bmatrix} hs_{re,1} & hs_{ac,1} & hs_{ou,1} \\ hs_{re,2} & hs_{ac,2} & hs_{ou,2} \\ \vdots & \vdots & \vdots \\ hs_{re,10} & hs_{ac,10} & hs_{ou,10} \end{bmatrix}$$

where:

- $hs_{re,i}$: The proportion of the resting state in the $i$-th time period; $hs_{ac,i}$: The proportion of the active state in the $i$-th time period; $hs_{ou,i}$: The proportion of the out state in the $i$-th time period.

Each row represents the proportion distribution of the three states in a specific time period.

#### Generation of the state matrix

**Definition of Upper and Lower Bounds for Date Attributes** For each time period, the upper and lower bounds for the resting and active states are defined based on the date type (e.g., working days, weekends, holidays). The matrix is as follows:

$$Ranges_{Datetype} = \{(hs_{re_{\text {down}, i}}, hs_{re_{\text {up}, i}}, hs_{ac_{\text {down}, i}}, hs_{ac_{\text {up}, i}}) \mid i = 1, 2 \ldots 10 \}$$

where:

- $hs_{ac_{\text {down},i}}$ and $hs_{ac_{\text {up},i}}$: The lower and upper bounds of the activity state proportion in the $i$-th time period;
- $hs_{re_{\text {up},i}} + hs_{ac_{\text {up},i}} < 1$: Ensures that the sum of the state proportions in each time period does not exceed 1.

**Random Generation of State Proportions** Within the defined upper and lower bounds, the resting and active state proportions for each time period are randomly generated. The out state proportion is determined by the complement:

$$\begin{aligned} & HSM_{hs_{re}, i} \sim \text {Uniform}(hs_{re_{downi}}, hs_{re_{upi}}) \\ & HSM_{hs_{ac}, i} \sim \text {Uniform}(hs_{ac_{downi}}, hs_{ac_{upi}}) \\ & HSM_{hs_{ou}, i} = 1 - HSM_{hs_{re}, i} - HSM_{hs_{ac}, i} \end{aligned}$$

where:

- $HSM_{hs_{re},i}$, $HSM_{hs_{ac},i}$, and $HSM_{hs_{ou},i}$: The proportions of resting, active, and away states in time period $i$, respectively, and their sum equals 1.

#### Generation of minute-level dynamic data

After the status matrix is generated, it is interpolated a minute-level time series. Combined with household numbers and family size, dynamic population data is generated.

**Timestamp Range Matrix:** The start and end times for each time period are defined. The time stamp matrix is as follows:

$$Ranges_{stamp} = \begin{bmatrix} stamp_{down_1} & stamp_{up_1} \\ stamp_{down_2} & stamp_{up_2} \\ \vdots & \vdots \\ stamp_{down_{10}} & stamp_{up_{10}} \end{bmatrix}$$

**Minute-Level Dynamic Data Interpolation:** For each family size, dynamic data is generated and interpolated:

$$RPDD_{fs} = \text {pchip}(Stamp_{fs}, HSM_{fs}, Time_{\text {series}}) \cdot nh_{fs} \cdot fs$$

where:

- $RPDD_{fs}$: Minute-level dynamic data for households of size $fs$; $nh_{fs}$: Number of households in the residential area;
- $fs$: Family size; $\text {Time}_{\text {series}}$: Minute-level time series.

Finally, the dynamic data for each residential area is combined to form the residential population dynamic model:

$$P_{rec} = \{ RPDD_{fs} \mid fs = 1,2,...,6 \}$$

### Dynamic modeling of personnel in administrative areas

The personnel dynamics in administrative areas are influenced by fixed work hours, and the modeling is based on predefined time nodes. Considering the changes in commuting numbers on different date types (weekdays, weekends, and holidays), this paper constructs a personnel status matrix for the administrative area and combines it with the residential area personnel dynamic model $P_{rec}$ and the corresponding timestamp matrix for modeling.

**Personnel Status Matrix for Administrative Areas:** The personnel status matrix for administrative areas is defined as follows:

$$PSM_{adm} = \begin{bmatrix} pa_{wd,1} & pa_{wd,2} & \cdots & pa_{wd, n} \\ pa_{we,1} & pa_{we,2} & \cdots & pa_{we, n} \\ pa_{hd,1} & pa_{hd,2} & \cdots & pa_{hd, n} \end{bmatrix}$$

where:

- $pa_{\text {wd},i}$: Personnel percentage at the $i$-th time point on weekdays; $pa_{\text {we},i}$: Personnel percentage at the $i$-th time point on weekends; $pa_{\text {hd},i}$: Personnel percentage at the $i$-th time point on holidays.

**Introduction of Random Fluctuations:** To simulate the randomness of personnel changes, a uniform distribution-based random fluctuation is applied to the personnel percentage at each time point, as given by:

$$pa_{i} = pa_{i} \cdot (1 + \text {Uniform}(\varepsilon _{ad}, \varepsilon _{au}))$$

where:

- $\varepsilon _{\text {ad}}$: Lower limit of the random fluctuation; $\varepsilon _{\text {au}}$: Upper limit of the random fluctuation.

**Generation of Minute-Level Dynamic Data:**

**Timestamp Matrix for Administrative Areas:**

The timestamp matrix for the administrative area $Ranges_{\text {stampadm}}$ is defined as:

$$Ranges_{stampadm} = [ stamp_{adm1}, stamp_{adm2}, \cdots , stamp_{admn} ]$$

where $\text {stamp}_{\text {admi}}$ is the timestamp of the $i$-th time point in the administrative area.

**Minute-Level Dynamic Data Interpolation:**

By interpolating the personnel status matrix of the administrative area, the administrative area personnel dynamic model $P_{adm}$ is obtained:

$$P_{adm} = \text {pchip}(Ranges_{stampadm}, PSM_{adm}, Time_{series}) \cdot P_{rec} \cdot \xi _{adm}$$

where $\xi _{\text {adm}}$ is Commuting coefficient from the residential area to the administrative area.

### Dynamic modeling of personnel in industrial areas

In industrial enterprises, there is no unified schedule for work shifts, and each enterprise independently determines its shift system. To simplify the modeling, the shift systems are categorized into two types: **three-shift systems** and **non-three-shift systems**. The characteristic of a three-shift system is that personnel rotate through shifts, including night shifts, with a constant number of personnel during each shift change. In contrast, the non-three-shift system is more flexible and dispersed according to production demands, leading to a broader range of working hours for employees.

**Dynamic Data for Three-Shift System Personnel:** Personnel in the three-shift system typically rotate through shifts, including night shifts, and the number of personnel per shift remains stable. In the model, the number of three-shift system personnel is related to the minimum number of people leaving the residential area and a fixed proportional coefficient $\alpha _{\text {sts}}$, calculated as:

$$P_{STS} = P_{\text {rec}, \text {ou}_{\text {min}}} \cdot \alpha _{sts}$$

where:

- $P_{\text {STS}}$: Three-shift system personnel dynamic data; $P_{\text {rec}, \text {ou}_{\text {min}}}$: Minimum number of residents leaving the residential area; $\alpha _{\text {sts}}$: Proportional coefficient representing the proportion of shift workers (mainly night shift workers) among the outbound personnel.

**Non-Three-Shift Personnel State Matrix:**

**Shift Allocation Proportions**

For non-three-shift personnel, there is no unified working schedule. Instead, their work hours are distributed across different time periods based on production requirements. The model divides the shift system of the industrial zone into 10 types, with each type’s personnel proportion denoted as $\beta _{\text {i}}$, and the sum of these proportions satisfies:

$$\sum _{i = 1}^{10} \beta _{ind} = 1$$

**Determination of Population Base**

1. The total population base of the industrial zone is determined by the total number of residents in the residential area and the commuting coefficient $\xi _{\text {ind}}$:
  $$P_{\text {baseline}, \text {ind}} = P_{\text {rec}} \cdot \xi _{ind}$$
2. After subtracting the three-shift personnel (where the number of personnel per shift is fixed), the baseline number of non-three-shift personnel is:
  $$P_{\text {baseline}, \text {NTS}} = P_{\text {baseline}, \text {ind}} - 3 \times P_{STS}$$
3. The baseline population value for the $k$-th type of shift system is:
  $$P_{\text {baseline}, k} = P_{\text {baseline}, \text {NTS}} \cdot \beta _k$$

**Non-Three-Shift Industrial Personnel Dynamic Matrix**

1. The dynamic state matrix for non-three-shift personnel is:
  $$PSM_{\text {ind}, k} = \begin{bmatrix} pi_{\text {wd},1, k}, pi_{\text {wd},2, k}, \dots , pi_{\text {wd}, n, k} \\ pi_{\text {we},1, k}, pi_{\text {we},2, k}, \dots , pi_{\text {we}, n, k} \\ pi_{\text {hd},1, k}, pi_{\text {hd},2, k}, \dots , pi_{\text {hd}, n, k} \end{bmatrix}$$
  where:
  $pi_{\text {wd},i,k}$: The percentage of personnel at time point $i$ for the $k$-th shift system on a weekday; $pi_{\text {we},i,k}$: The percentage of personnel at time point $i$ for the $k$-th shift system on weekends; $pi_{\text {hd},i,k}$: The percentage of personnel at time point $i$ for the $k$-th shift system on holidays.
2. Introduction of Random Fluctuations: To model the random fluctuations in personnel flow, the following random disturbance is introduced:
  $$pi_{i} = pi_{i} \cdot (1 + \text {Uniform}(\varepsilon _{id}, \varepsilon _{iu}))$$
  where:
  $\varepsilon _{id}$: The lower limit of the random fluctuation; $\varepsilon _{iu}$: The upper limit of the random fluctuation.
3. Non-Three-Shift Time Stamp Matrix: The set of time stamps for the multi-shift systems in the industrial zone, denoted as $Ranges_{\text {stampind}}$, is defined as:
  $$Ranges_{\text {stampind}, k} = \{ [ stamp_{\text {ind1}, k}, stamp_{\text {ind2}, k} \dots stamp_{\text {indn}, k}] \mid k = 1,2 \dots 10 \}$$
  where $stamp_{\text {indi},k}$ represents the time stamp at time point $i$ for the $k$-th shift system.
4. Minute-Level Dynamic Data Interpolation: Based on the industrial zone personnel state matrix, minute-level dynamic data is generated using interpolation:
  $$IPDD_{k} = \text {pchip}(Ranges_{\text {stampind}}, PSM_{\text {ind}, k}, Time_{\text {series}}) \cdot P_{\text {baseline}, k}$$
  where $P_{\text {baseline}, k}$ is the baseline population value for the $k$-th shift system.

**Non-Three-Shift Personnel Dynamic Data:**

$$P_{\text {NTS}} = \sum _{k = 1}^{10} IPDD_k$$

**Final Industrial Zone Personnel Dynamic Data:** The final industrial zone personnel dynamic model is obtained by combining the three-shift personnel and non-three-shift personnel data:

$$P_{\text {ind}} = P_{STS} + P_{NTS}$$

### Dynamic modeling of personnel in commercial areas

The population dynamics in commercial areas exhibit high randomness, with significant differences in patterns compared to administrative and industrial zones. To construct a reasonable population dynamic model for commercial areas, the following assumptions are made:

- **Spatial Distance and Commuting Time**: The population movement must consider the spatial distance between residential areas and functional zones (e.g., administrative, industrial, and commercial areas), with commuting time used as a representation.
- **Dynamic Increment Modeling**: The dynamic population change is described by the incremental change in population per unit of time (e.g., per minute).
- **Initial Conditions and Random Fluctuations**: The population distribution in the commercial area is set based on initial values, and a random fluctuation adjustment mechanism is introduced.

#### Dynamic increment modeling of population

**Dynamic Increment for Administrative and Industrial Areas:** The dynamic increment for the administrative and industrial areas is calculated as:

$$\Delta P_{adm} = P_{adm}(t)-P_{adm}(t-1) \quad \Delta P_{ind} = P_{ind}(t)-P_{ind}(t-1)$$

**Residential Area Outbound Population Increment:**

$$\Delta P_{res, ou}= P_{res, ou}(t)-P_{res, ou}(t-1)$$

**Increment Shift (Commuting Time):** Based on the commuting time range, the time offset for the population dynamic increment from administrative and industrial areas (either arriving or departing) is calculated as:

$$\begin{aligned} \Delta P'_{adm} (t_i)= & \Delta P_{adm}(t_i) \\ t_i'= & t_i + \delta t_i' \\ \delta t_i'= & {\left\{ \begin{array}{ll} -|\delta t_i| & \text {if} \, \Delta P_{adm}(t_i)> 0 \\ +|\delta t_i| & \text {if} \, \Delta P_{adm}(t_i) < 0 \end{array}\right. } \\ \delta t_i\in & [D_{rza, down}, D_{rza, up}] \end{aligned}$$

where:

- $D_{rza, \text {down}}$ and $D_{rza, \text {up}}$: The lower and upper bounds of the commuting time range, respectively.

Similarly, the adjustment for the industrial area increment is:

$$\Delta P'_{ind} (t_i) = \Delta P_{ind}(t_i)$$

**Dynamic Increment of Population in Commercial Areas:** In the outbound population dynamic increment from the residential area, the portion directed toward administrative and industrial zones is excluded, resulting in the corrected outbound increment for the residential area:

$$\Delta P'_{res, ou}=\{ \Delta P_{res, ou}-\left( \Delta P'_{adm} (t_i)+\Delta P'_{ind} (t_i)\right) \}$$

Based on the corrected outbound increment from the residential area, the dynamic increment of the commercial area population is calculated as:

$$\Delta P_{com}(t)=\Delta P'_{res, ou} (t) \cdot \xi _{com} \cdot \text {Uniform}(\varepsilon _{com_{down}}, \varepsilon _{com_{up}})$$

where:

- $\xi _{com_j}$: The commuting coefficient from residential areas to commercial areas;
- $\varepsilon _{com_{j, down}}$ and $\varepsilon _{com_{j, up}}$: The upper and lower bounds for dynamic fluctuations in population migration to commercial areas.

#### Dynamic data generation

**Initial Population Migration Conditions:**

$$P_{com}(1)= P_{res, ou}(1) \cdot \alpha _{com}$$

The dynamic model for the population in commercial areas is obtained by summing the population migration increments for each residential area:

$$P_{com}= P_{com}(1)+\sum _{t = 1}^{t_{end}}\Delta P_{com}(t)$$

## Distribution network non-controlled load model

The distribution network non-controlled load model is an essential component of intelligent power load management, providing energy consumption reference. This study constructs non-controlled load models for residential and functional areas based on the load characteristics in different zones within the distribution network. After removing the impact of air conditioning loads, this model serves as a benchmark for verifying the accuracy and practicality of air conditioning load modeling.

### Non-controlled load classification and modeling strategy

Non-controlled loads are categorized into **residential non-controlled load** and **functional area non-controlled load**, with modeling strategies tailored to the characteristics of each area:

- **Residential Non-Controlled Load**: The non-controlled load in residential areas exhibits significant aggregation characteristics, with different load types dominating during specific time periods. For instance, kitchen load dominates during mealtime periods, electronic equipment load predominates after work hours, and standby load dominates at night^31^. Studies show that residents’ activity patterns are closely related to the frequency of appliance usage, particularly as appliance usage frequency varies with the residents’ activity status during different time periods^32^. Therefore, this paper constructs a dynamic usage frequency model combined with the home state matrix to simulate the fluctuation characteristics of residential non-controlled loads.
- **Functional Area Non-Controlled Load**:
  - **Administrative Load**: This type of load generally exhibits a stable average power.
  - **Industrial Load**: Industrial loads have higher power levels due to varying production processes and fluctuate according to production patterns.
  - **Commercial Load**: The load in commercial areas is complex and difficult to decompose into a single load object.

### Residential non-controlled load model

Residential loads are categorized into six major types:

$$\text {Load Categories: } \{ \text {SBL, IL, EEL, KL, LL, OL} \}$$

where the definitions of load types are as follows:

- **SBL**: Standby Load (e.g., refrigerators, freezers, TVs)
- **IL**: Instantaneous Load (e.g., water heaters)
- **EEL**: Electronic Equipment Load (e.g., computers, TVs)
- **KL**: Kitchen Load (e.g., ovens, microwave ovens)
- **LL**: LED Lighting Load (e.g., LED lamps)
- **OL**: Other Loads (e.g., miscellaneous small appliances)

Each load type corresponds to a set of typical devices:

$$\mathscr {D}_\text {SBL}, \mathscr {D}_\text {IL}, \mathscr {D}_\text {EEL}, \mathscr {D}_\text {KL}, \mathscr {D}_\text {LL}, \mathscr {D}_\text {OL}$$

For example, the standby load (SBL) device set $\mathscr {D}_\text {SBL}$ includes devices such as refrigerators, freezers, and televisions.

#### Device power dataset

Each device’s rated power dataset, $\mathscr {P}_d$ represents the possible rated power values for device $d$. For example, the power dataset for a refrigerator is:

$$\mathscr {P}_\text {Fridge} = \{ 70, 90, 100, 130, 150, 170, 180, 200 \} \; (\text {W})$$

The full set of rated powers for all devices is:

$$\mathscr {P}_\text {SBL} = \bigcup _{d \in \mathscr {D}_\text {SBL}} \mathscr {P}_d\quad \mathscr {P}_\text {IL} = \bigcup _{d \in \mathscr {D}_\text {IL}} \mathscr {P}_d \dots \quad \mathscr {P}_\text {OL} = \bigcup _{d \in \mathscr {D}_\text {OL}} \mathscr {P}_d$$

For standby load (SBL), the typical devices and power set are represented as:

$$\mathscr {D}_\text {SBL} = \{ \text {Fridge, Freez, TV, Rtr, OSD} \}$$

The power set is as follows:

$$\mathscr {P}_\text {Fridge} = \{ 70, 90, 100, 130, 150, 170, 180, 200 \} \; (\text {W})$$

$$\mathscr {P}_\text {Freez} = \{ 50, 70, 90, 120, 140, 160, 180, 200 \} \; (\text {W})$$

#### Power distribution modeling

For each device, an appropriate probability distribution is chosen to simulate the fluctuation of its power values.The probability distribution types used in the model are detailed in Table 3.

**Table 3:** Probability Distribution Types.

Distribution Type	Chosen Probability Distribution
Front Heavy Distribution	[0.25, 0.3, 0.2, 0.15, 0.04, 0.03, 0.02, 0.01]
Mid-front Heavy Distribution	[0.1, 0.35, 0.3, 0.15, 0.04, 0.03, 0.02, 0.01]
Mid Heavy Distribution	[0.02, 0.08, 0.1, 0.3, 0.3, 0.1, 0.08, 0.02]
Mid-rear Heavy Distribution	[0.01, 0.02, 0.03, 0.04, 0.15, 0.3, 0.35, 0.1]
Rear Heavy Distribution	[0.01, 0.02, 0.03, 0.04, 0.1, 0.15, 0.3, 0.35]
Uniform Distribution	[0.125, 0.125, 0.125, 0.125, 0.125, 0.125, 0.125, 0.125]

#### Family size dependence of power distribution

Family Size influences the selected power for devices in two main ways:

- As the Family population increases, device demand and power levels exhibit an increasing trend.
- The probability distribution for device power selection varies significantly across different Family sizes.

**Average Load Power:**

The average load power $\text {ALP}_L(fz)$ is calculated as:

$$\text {ALP}_L(fz) = \sum _{d \in \mathscr {D}_L} \left( \mathscr {P}_d \cdot \textbf{PDS}(fz) \right)$$

where:

- $L$ denotes the load type;
- $fz$ represents the household size;
- $\mathscr {D}_L$ is the device set for load type $L$;
- $PDS(fz)$ is the power distribution corresponding to the household size $fz$.

#### Dynamic usage frequency modeling

The frequency of usage is related to the home state matrix, where the frequency of device usage varies with the three possible home states: resting, active, and away. The state correlation matrix $\textbf{R}_L$ for load type $L$ consists of Boolean elements representing the relationship between the load type and the state. For instance:

$$R_{SBL} = \begin{bmatrix} 1 \\ 0 \\ 1 \end{bmatrix} \quad R_{LL} = \begin{bmatrix} 1 \\ 0 \\ 0 \end{bmatrix} \quad R_{OL} = \begin{bmatrix} 0 \\ 1 \\ 0 \end{bmatrix}$$

To describe device usage frequency, two sets are defined: one for the timestamp range set (Timestamp Range Set) $\text {TRS}_L$ and the other for the usage probability set (Usage Probability Set) $\text {UPS}_L$.

For each load type $L$, the timestamp range set $\text {TRS}_L$ and usage probability set $\text {UPS}_L$ are defined as:

$$\begin{aligned} \text {TRS}_L= & \{ [t_{d,1}, t_{u,1}], [t_{d,2}, t_{u,2}] \dots [t_{d, n}, t_{u, n}] \} \\ \text {UPS}_L= & \{ fr_1, fr_2 \dots fr_n \} \\ t_{i} \sim \text {Uniform}(t_{d, i}, t_{u, i}) \mid i= & 1,2,\dots ,n \\ \text {UTPS}_L= & \{t_1, t_2 \dots t_n\} \end{aligned}$$

At each timestamp, a set of usage time points $\text {UTPS}_L$ is generated randomly and interpolated to minute-level discrete points using the piecewise cubic Hermite interpolating polynomial (PCHIP), resulting in the minute-level usage probability set $\text {ISUC}_L$:

$$\text {ISUC}_L(t) = \text {pchip}(\text {UTPS}_L, \text {UPS}_L, \text {Time}_{\text {series}})$$

The dynamic usage frequency data $DU_L(t)$ is calculated as:

$$DU_L(t) = R_L \cdot HSEM(hs) \cdot \text {ISUC}_L(t)$$

**Residential Non-Controlled Load:**

$$L_{RNC}(t) = \sum _{fz = 1}^6 DU_L(t) \cdot \text {ALP}_L(fz) \cdot (1 + \varepsilon _{rt})$$

where:

- $fz$ is the family size;
- $\varepsilon _t$ is the dynamic fluctuation term, with $\varepsilon _{t_d}$ and $\varepsilon _{t_u}$ representing the lower and upper bounds of the fluctuation term.

### Non-regulated load model of functional zones

The load types in functional zones are complex and diverse, making it difficult to analyze their composition individually. Therefore, a simplified mean value modeling approach is adopted in this study to construct the load model for functional zones, providing data support for further analysis and regulation.

#### Administrative Load

The dynamic load of the administrative area is based on the timestamp set of personnel flow, $Ranges_{\text {stampadm}}$, and the corresponding per capita load set, $PCL_{\text {adm}}$ (Per Capita Load Set by Administrative Area). Interpolation is used to extend the data. The load model is given by the following equation:

$$L_{adm}(t) = \text {pchip}(Ranges_{\text {stampadm}}, PCL_{\text {adm}}, \text {Time}_{\text {series}}) \cdot (1 + \varepsilon _{at})$$

where:

- $\varepsilon _{at}$ represents the fluctuation term of the administrative zone load, which accounts for load variations due to personnel dynamics and external factors such as weather changes.

#### Industrial load

The modeling of the industrial load considers that during night shifts or overtime, the number of operators may decrease, but the production lines continue to operate. To model this, the mean value method is used, and the load of the industrial zone is calculated by inversely mapping the dynamic personnel count. The load model is expressed as:

$$L_{ind}(t) = {\left\{ \begin{array}{ll} L_{\text {min}} & \text {if } t = T_{\text {max}} \\ L_{\text {max}} & \text {if } t = T_{\text {min}} \\ L_{\text {min}} + (L_{\text {max}} - L_{\text {min}}) \cdot \frac{P_{ind}(t) - P_{\text {indmin}}}{P_{\text {indmax}} - P_{\text {indmin}}} & \text {otherwise} \end{array}\right. }$$

where:

- $T_{\text {max}}$ denotes the index for the maximum value;
- $T_{\text {min}}$ denotes the index for the minimum value;
- $P_{\text {indmin}}$ and $P_{\text {indmax}}$ represent the minimum and maximum personnel flow values in the industrial area;
- $L_{\text {max}}$ and $L_{\text {min}}$ are the maximum and minimum industrial load values.

#### Commercial load

The load in the commercial area exhibits significant temporal dependence, especially during peak meal times when the load increases. At night, the load also increases as lighting and personnel in entertainment venues grow. Thus, the model is constructed using an extended time point set, $Ranges_{T_{\text {com}}}$, and the corresponding average load set, $Ranges_{L_{\text {com}}}$. The load model is formulated as:

$$L_{com}(t) = P_{com} \cdot \text {pchip}(Ranges_{T_{\text {com}}}, Ranges_{L_{\text {com}}}, \text {Time}_{\text {series}}) \cdot (1 + \varepsilon _{ct})$$

where:

- $\varepsilon _{ct}$ represents the fluctuation term of the commercial zone load, which reflects the impact of factors such as commercial peak periods and personnel flow.

## Indoor temperature model

The indoor temperature is a key variable for determining the operational state of AC units. This model is based on the principle of heat balance, incorporating factors such as solar radiation, heat transfer between indoor and outdoor environments, heat generated by human activities, and heat loss related to wind speed^33^. Studies have shown that the impact of solar radiation on indoor temperature depends not only on window orientation and area but also on the building’s thermal mass and ventilation conditions^34^. Additionally, heat dissipation from human activities constitutes an essential part of the indoor heat load, with the amount of heat generated closely related to the intensity and state of the activity^35^. By constructing a heat balance model and integrating meteorological data and human dynamics, the variations in indoor temperature are accurately simulated.

### Heat balance model

Indoor temperature variations are governed by the balance between heat input and output. Using discrete time steps, the numerical calculation of indoor temperature is expressed as:

$$T_{\text {indoor}}(t + \Delta t) = T_{\text {indoor}}(t) + \frac{\Delta t}{k_c \cdot C_{\text {indoor}}} \cdot (Q_{\text {in}} - Q_{\text {loss}})$$

where:

- $C_{\text {indoor}}$: Total thermal capacity of the air-conditioned room (J/K), determined by room volume and material properties;
- $k_c$: Thermal capacity coefficient, the values are shown in Table 10;
- $Q_{\text {in}}$: Heat input, including solar radiation and heat generated by human activities;
- $Q_{\text {loss}}$: Heat loss, including heat transfer and wind speed-related dissipation.

### Heat input

Solar radiation is a primary source of indoor heat, influenced by factors such as window orientation, area, and solar radiation intensity^36^. Research indicates that window orientation and solar intensity significantly affect the distribution of indoor temperatures, especially as solar heat input fluctuates with changing sun angles over time^37^. Additionally, heat generated by human activities forms a crucial part of the indoor heat load, with the heat dissipation closely tied to the intensity and state of resident activity^35^. Therefore, solar radiation and human activity heat input processes are simulated using corresponding models, supported by meteorological and human dynamics data.

#### Solar radiation heat input

Solar radiation heat input is modeled as follows:

$$Q_{\text {solar}} = A_{\text {window}} \cdot I_{\text {solar}} \cdot f_{\text {angle}}$$

where:

- $A_{\text {window}}$: Window area ($\hbox {m}^{2}$), determined by building design;
- $I_{\text {solar}}$: Solar radiation intensity per unit area ($\hbox {W}/\hbox {m}^{2}$), generated via interpolation of meteorological data;
- $f_{\text {angle}}$: Orientation correction factor, dependent on window orientation and solar angle.

#### Human activity heat generation

Heat generated by residents depends on activity intensity and living conditions, modeled as follows:

$$Q_{\text {human}} = \text {PDM}_{\text {act}} \cdot P_{\text {activity}}$$

where:

- $\text {PDM}_{\text {act}}$: Activity state count derived from the dynamic personnel model;
- $P_{\text {activity}}$: Heat dissipation per individual (W), determined based on resident activity curves and randomized factors.
  - Resting state: Approximately 70 W.
  - Light activity: Approximately 100 W.
  - Intense activity: Approximately 200 W.

#### Indoor-outdoor heat transfer

Heat transfer between indoor and outdoor environments contributes to the total heat input, depending on the temperature difference and building thermal properties^38^. This term accounts for heat gain when outdoor temperatures exceed indoor levels, and heat loss in the opposite scenario, integrated into the input framework as requested by reviewer feedback. The model is:

$$Q_{\text {conductive}} = k_a \cdot A_{\text {exposed}} \cdot (T_{\text {outdoor}} - T_{\text {indoor}})$$

where:

- $A_{\text {exposed}}$: Total exposed area of external walls and windows ($\hbox {m}^{2}$), generated randomly;
- $T_{\text {outdoor}}$: Outdoor temperature from meteorological data;
- $k_a$: Heat transfer coefficient $(\hbox {W}/\hbox {m}^{2}\cdot \hbox {K})$, randomly generated based on the thermal insulation properties of building materials.

A positive $Q_{\text {conductive}}$ indicates heat inflow from the outdoor environment, while a negative value reflects heat loss, both incorporated into the total heat input.

#### Fresh air heat gain

Fresh air intake introduces additional heat gain, particularly in air-conditioned spaces, as outdoor air exchanges with indoor air through ventilation or infiltration. This term is included to address reviewer feedback on the omission of fresh air effects. The heat gain due to fresh air is modeled as:

$$Q_{\text {fresh}} = \dot{m}_{\text {fresh}} \cdot c_p \cdot (T_{\text {outdoor}} - T_{\text {indoor}})$$

where:

- $\dot{m}_{\text {fresh}}$: Mass flow rate of fresh air (kg/s), set to a typical value of 0.005 kg/s for minimal ventilation in closed rooms;
- $c_p$: Specific heat capacity of air ($\hbox {kJ}/\hbox {kg}\cdot \hbox {K}$), approximately 1.005 $\hbox {kJ}/\hbox {kg}\cdot \hbox {K}$;
- $T_{\text {outdoor}} - T_{\text {indoor}}$: Temperature difference between outdoor and indoor air (°C).

This term contributes positively to heat input when outdoor temperatures exceed indoor levels, with its small magnitude ensuring minimal disruption to the overall model, as validated in simulations.

The total heat input is updated as:

$$Q_{\text {in}} = k_s \cdot Q_{\text {solar}} + k_p \cdot Q_{\text {human}} + Q_{\text {conductive}} + Q_{\text {fresh}}$$

where:

- $$k_s$$: Solar coefficient;
- $k_p$: Human heat coefficient.

### Heat loss

Heat loss is primarily influenced by wind speed-related dissipation, exacerbated by outdoor wind speeds and building ventilation areas^40^. Studies suggest that building thermal mass and ventilation conditions significantly affect heat transfer efficiency, with higher wind speeds increasing dissipation^36^. The heat loss process is simulated using wind-related models supported by meteorological and building-specific data. Regarding ventilation effects, the model implicitly accounts for natural ventilation through the wind speed and exposed area parameters, as these factors capture the convective heat exchange driven by outdoor air movement. Mechanical ventilation, such as forced air exchange via air-conditioning systems, is not separately modeled here, as its primary thermal contribution is addressed in the heat input section through fresh air heat gain. This approach simplifies the heat loss formulation while maintaining consistency with the overall energy balance, though future refinements could explicitly incorporate mechanical ventilation effects if detailed system data were available.

#### Wind speed-related heat dissipation

Wind speed accelerates heat exchange between indoor and outdoor environments, increasing heat loss. The wind-related heat dissipation model is:

$$Q_{\text {wind}} = v_{\text {wind}} \cdot A_{\text {exposed}}$$

where:

- $A_{\text {exposed}}$: Ventilation area matrix ($\hbox {m}^{2}$), generated randomly based on indoor area, representing the effective area for natural convective exchange;
- $v_{\text {wind}}$: Outdoor wind speed (m/s) from meteorological data, driving both natural ventilation and associated heat dissipation.

This formulation integrates the effects of wind-induced natural ventilation, with $A_{\text {exposed}}$ reflecting potential openings such as windows or vents, while mechanical ventilation impacts are subsumed under the fresh air heat gain term in the heat input model.

The total heat loss is:

$$Q_{\text {loss}} = k_{\text {loss}} \cdot Q_{\text {wind}}$$

where:

- $k_{\text {loss}}$: Wind heat dissipation coefficient, generated based on the building environment, encapsulating the efficiency of convective heat loss.

**Fig. 2:**
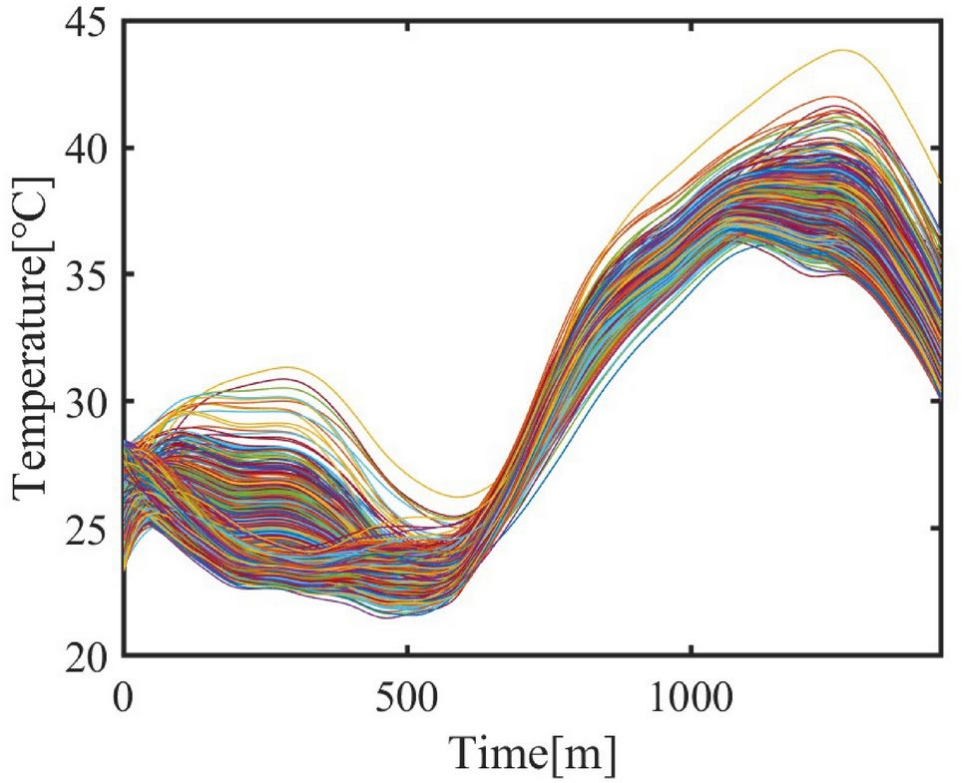
Indoor Temperature Variations Over 24 Hours.

The indoor temperature variations over 24 hours for all air-conditioned rooms, reflecting the updated heat input and loss models, are depicted in Figure 2, exhibiting diverse profiles while demonstrating a general trend tied to the variability of room heat balance and correlation with outdoor temperature fluctuations.

## Air conditioning operation signal model

The on/off state of air conditioners is influenced not only by temperature regulation strategies but also by residents’ activity states and thermal comfort requirements^38^. Studies show that residents’ tolerance to temperature fluctuations and AC usage habits significantly affect the operating status of air conditioners, particularly in different time periods, where activation frequency and duration vary with activity states^40^. Therefore, this study constructs threshold temperature and tolerance time models, integrating residents’ activity states and AC usage habits, to simulate the operational status of air conditioners.

### Generation of threshold temperature and tolerance time

By generating threshold temperatures and tolerance times with statistical characteristics, the model reflects diverse AC usage habits, effectively simulating load fluctuations.

#### Threshold temperature generation

The indoor temperature threshold for activating air conditioners typically ranges from ${26}^{\circ }$C to ${29}^{\circ }$C, with ${26.5}^{\circ }$C being the most common value. To simulate this phenomenon, a triangular distribution is adopted, with the peak set at ${26.5}^{\circ }$C to emphasize the concentrated nature of the temperature distribution. The generation formula is as follows:

$$T_{\textrm{thresh}, i} = {\left\{ \begin{array}{ll} T_{\min } + \sqrt{2 \cdot u \cdot (T_{\max } - T_{\min }) \cdot (T_{\text {peak}} - T_{\min })}, & u < 0.5 \\ T_{\max } - \sqrt{2 \cdot (1 - u) \cdot (T_{\max } - T_{\min }) \cdot (T_{\max } - T_{\text {peak}})}, & u \ge 0.5 \end{array}\right. }$$

where:

- $u \sim U(0, 1)$: A random number uniformly distributed between 0 and 1.

#### Tolerance time generation

When the indoor temperature reaches the set threshold, the user’s tolerance to temperature changes determines the delay before AC is activated. The tolerance time usually ranges from 0 to 15 minutes, with the most common value being 7.5 minutes. A triangular distribution is used, with the peak value set as $Tol_{\text {peak}}$ and the range defined as $[Tol_{\min }, Tol_{\max }]$. The generation formula is:

$$Tol_{\textrm{time}, i} = {\left\{ \begin{array}{ll} \sqrt{2 \cdot v \cdot Tol_{\max } \cdot Tol_{\text {peak}}}, & v < 0.5 \\ Tol_{\max } - \sqrt{2 \cdot (1 - v) \cdot Tol_{\max } \cdot Tol_{\text {peak}}}, & v \ge 0.5 \end{array}\right. }$$

where:

- $v \sim U(0, 1)$: A uniformly distributed random number.

### 0–1 state matrix modeling

The on/off state of air conditioners is not only governed by temperature regulation strategies but is also closely related to residents’ activity states, air conditioner configurations, and usage habits. Thus, modeling the operational state of air conditioners requires a comprehensive consideration of the dynamic interactions among these factors. This section details the modeling process, focusing on temperature control mechanisms, residents’ activity states, AC units, and their dynamic adjustments.

#### Step 1: initial on/off state

**Fig. 3:**
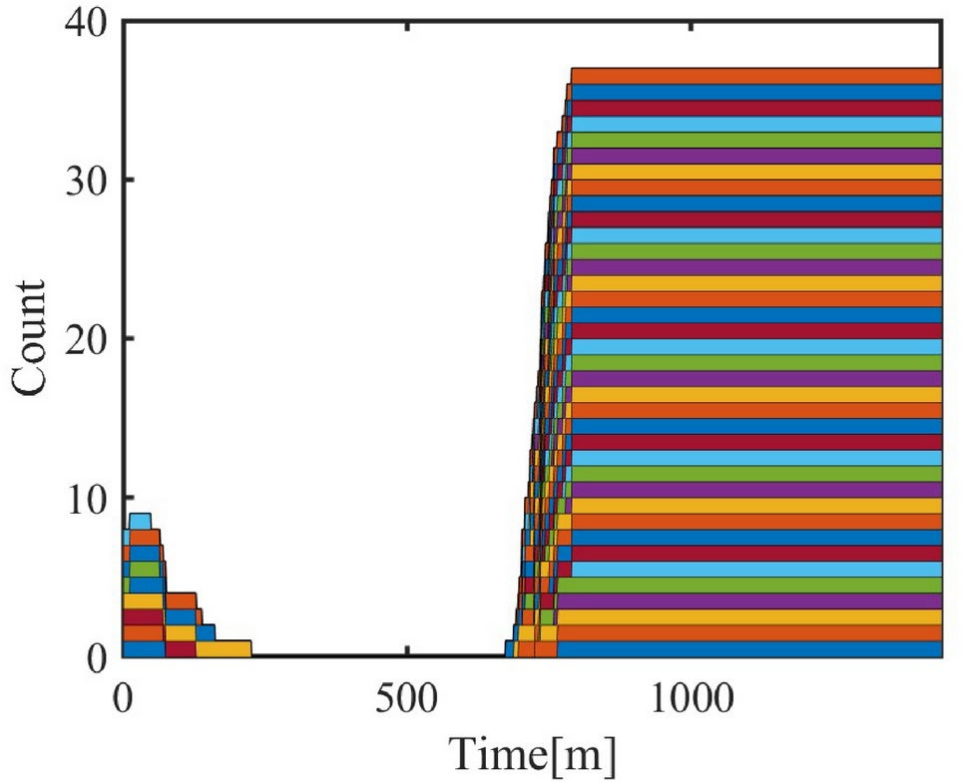
Living Room Air Conditioning 0–1 State Matrix (Step 1).

**Fig. 4:**
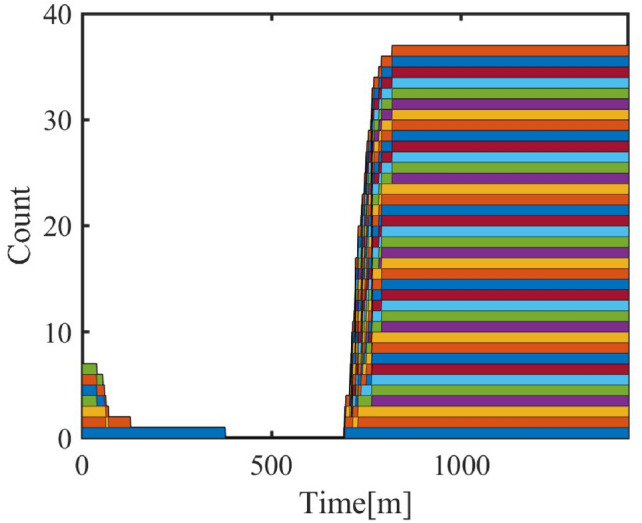
Living Room Air Conditioning 0–1 State Matrix (Step 2).

**Fig. 5:**
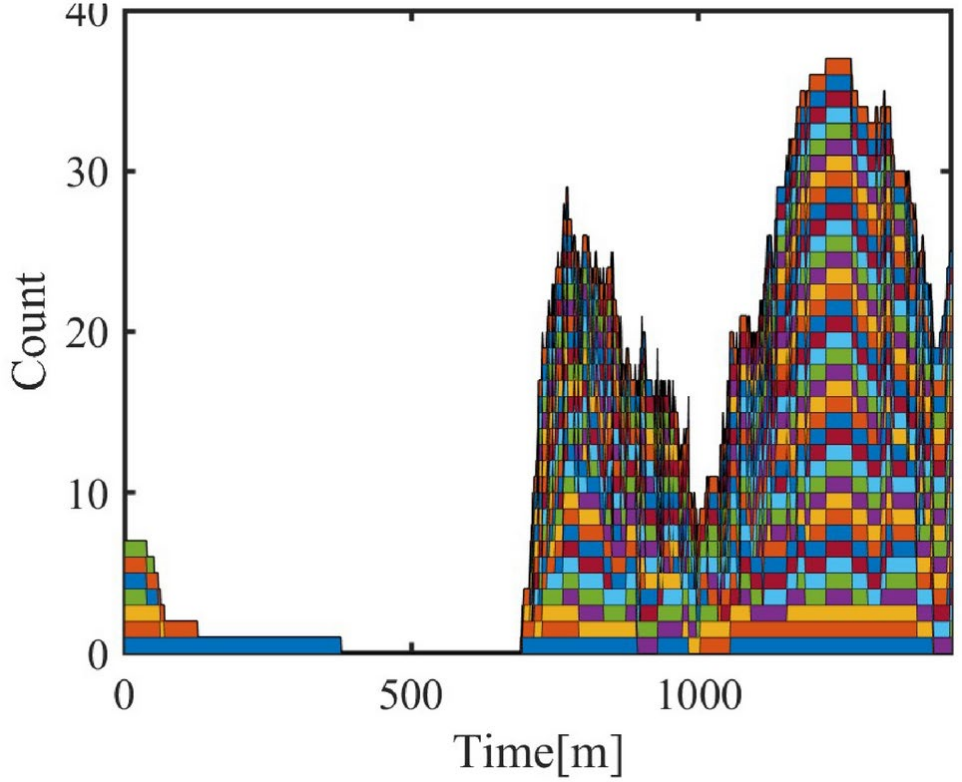
Living Room Air Conditioning 0–1 State Matrix (Step 4).

**Fig. 6:**
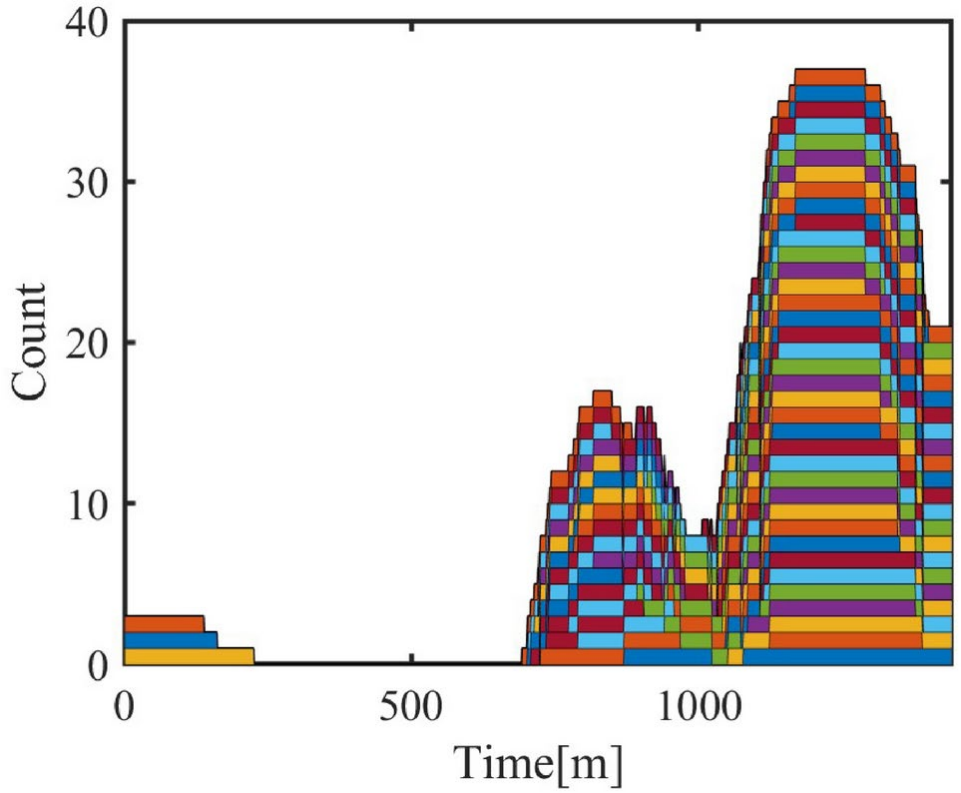
Living Room Air Conditioning 0–1 State Matrix (Step 5).

**Fig. 7:**
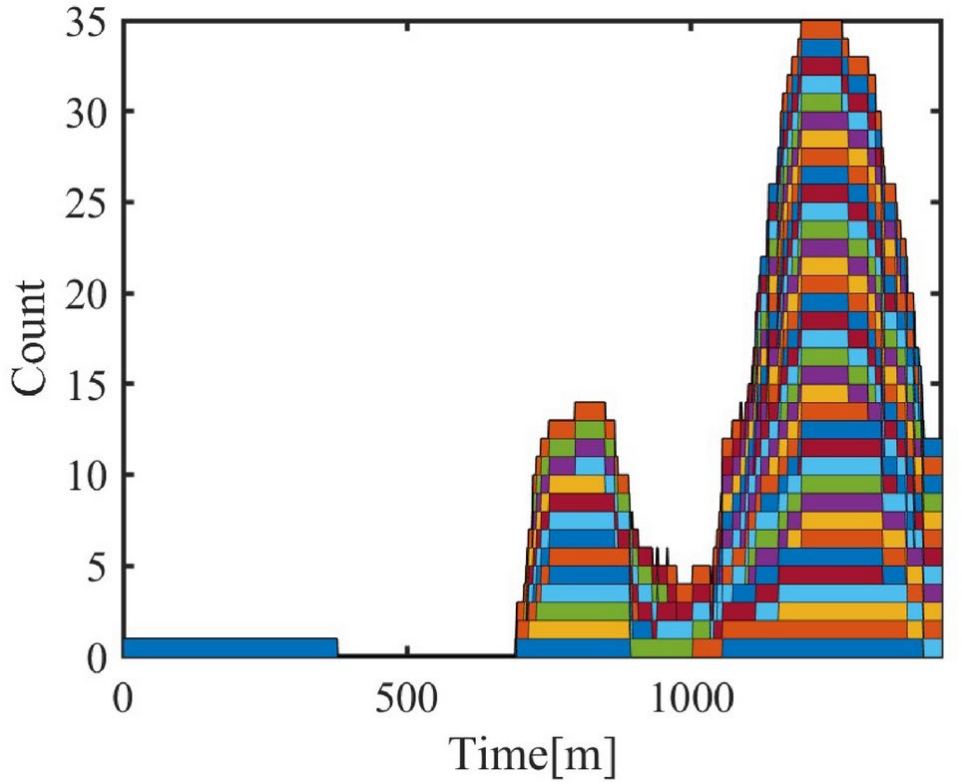
Living Room Air Conditioning 0–1 State Matrix (Step 6).

The preliminary on/off state $C(t)$ is determined based on the threshold temperature $T_{\text {thresh}}(t)$ and the indoor temperature $T_{\text {in}}(t)$. This matrix represents the air conditioner state at different time points, where 1 indicates “on” and 0 indicates “off”.The 0–1 state area charts corresponding to the steps are shown in Figures 3, 4, 5, 6 and 7.

#### Step 2: removal of short-term activation

If the activation duration of any air conditioner is less than the minimum sustained time $\Delta T_{\min }$, these short-term activations are considered invalid and set to 0 to avoid unreasonable fluctuations.

#### Step 3: dynamic scaling based on room characteristics and activities

Residents’ activity states directly affect the number of activated air conditioners. Under different activity states (e.g., resting, working, or being away), the on/off state of air conditioners changes significantly. The scaled activation state is adjusted dynamically using the personnel dynamic model:

$$C_{\textrm{scaled}}(t) = C_{\textrm{filtered}}(t) \times (1 + w_A \cdot \text {PDM} \cdot N_{\textrm{AC}}(t))$$

where:

- $w_A$: Activity intensity weight;
- $\text {PDM}(t)$: Personnel dynamic model, including $P_{rec}$, $P_{adm}$, $P_{ind}$, and $P_{com}$ to reflect activity intensities;
- $N_{\text {AC}}(t)$: Number of air conditioners.

#### Step 4: state matrix optimization with inheritance adjustment

To avoid excessive fluctuations, the scaled matrix is optimized using an inheritance mechanism. The current state is fused with the previous state using a weighting factor $\lambda$:

$$C_{\textrm{optimized}}(t) = C_{\textrm{scaled}}(t) + \lambda \cdot C_{\textrm{scaled}}(t - 1)$$

where:

- $\lambda$: Inheritance coefficient, controlling the influence of the previous state on the current state.

#### Step 5: repairing and connecting discontinuous states

After optimization, discontinuities in the on/off state (e.g., short “0” or “1” states) may still exist, particularly during significant changes in activity intensity. Short “off” states are repaired to “on” based on a repair threshold $\Delta T_{\text {repair}}$:

$$C_{\text {repaired}}(t) = {\left\{ \begin{array}{ll} 1 & \text {if duration}(C_{\text {optimized}}(t)) < \Delta T_{\text {repair}} \\ C_{\text {optimized}}(t) & \text {otherwise} \end{array}\right. }$$

where:

- $\Delta T_{\text {repair}}$: Time threshold for repairing.

#### Step 6: filtering short-term states again

After the repair, the matrix is filtered to remove newly generated short-term states using a threshold $\Delta T_{\text {filter}}$.

Through these steps, the AC operation signal model effectively simulates dynamic on/off states by integrating activity patterns, environmental temperatures, and the number of air conditioners, accurately reflecting the characteristics of AC load dynamics.

## Air conditioning system power modeling

Power modeling of air conditioning systems is a core technology in intelligent load control, encompassing power calculation, energy efficiency correction, cooling capacity adjustment, and dynamic temperature updates. These modules work collaboratively to simulate operational states and energy consumption characteristics of air conditioning systems, providing a reference for practical control strategies.

### Power calculation model

The total power consumption of an air conditioning system comprises compressor power and fan power, both dynamically influenced by indoor-outdoor temperature differences and setpoint deviations^39^. Studies indicate that outdoor temperature variations significantly affect the coefficient of performance (COP) and operational efficiency, with efficiency declining under high-temperature conditions^40^. To enhance realism, the compressor power model was revised to base its output primarily on the indoor-outdoor temperature difference ($\Delta T_{\text {c}}$), with dynamic adjustments driven by the setpoint temperature difference ($\Delta T_{\text {set}}$), as updated per reviewer feedback. This study constructs a power calculation model coupled with energy efficiency corrections and dynamic temperature updates to accurately simulate energy consumption characteristics under varying conditions.

#### Outdoor temperature difference

The outdoor temperature difference, defined as the deviation between the outdoor temperature $T_{\text {out}}$ and the indoor temperature $T_{\text {in}}$:

$$\Delta T_{\textrm{c}} = T_{\text {out}} - T_{\text {in}}$$

determines the baseline compressor power and influences both the COP and fan power demand. A larger $\Delta T_{\textrm{c}}$ increases the baseline power required to overcome environmental thermal loads, reflecting the system’s response to external conditions. This foundational parameter drives the initial power calculation, subsequently adjusted by setpoint considerations.

#### Setpoint temperature difference

The setpoint temperature difference, defined as the deviation between the current indoor temperature $T_{\text {in}}$ and the setpoint temperature $T_{\text {set}}$:

$$\Delta T_{\text {set}} = T_{\text {in}} - T_{\text {set}}$$

dynamically adjusts the compressor power through a control factor. Larger $\Delta T_{\text {set}}$ values increase power output to align the indoor temperature with the setpoint, while smaller values reduce power to maintain stability, ensuring responsive and efficient temperature regulation.

#### Compressor power

The compressor power is modeled based on the indoor-outdoor temperature difference ($\Delta T_{\textrm{c}}$), with adjustments driven by the setpoint temperature difference ($\Delta T_{\text {set}}$). The baseline compressor power is calculated as:

$$P_{\text {comp, base}} = P_{\text {max}} \cdot \left( a + b \cdot \frac{|\Delta T_{\textrm{c}}|}{\Delta T_{\text {max}}} \right)$$

where:

- $P_{\text {max}}$: Maximum compressor power;
- $a$: Base power coefficient;
- $$b$$: Scaling coefficient;
- $\Delta T_{\text {max}}$: Maximum allowable temperature difference.

The final compressor power incorporates a nonlinear control factor to enhance temperature regulation:

$$P_{\text {comp}} = P_{\text {comp, base}} \cdot \left[ \left( C_{f1} + \frac{\Delta T_{\text {set}}}{\Delta T_{\text {max}}} \right) ^2 - C_{f2} \right] \cdot S_{\text {fan}}$$

where:

- $C_{f1}, C_{f2}$: Coefficients of the nonlinear control function;
- $S_{\text {fan}}$: Switch signal (0 or 1), determined by operational state.

The control factor is constrained to [0, 5], allowing greater power scaling compared to the previous linear formulation, which was limited to [0, 1]. This revised model better captures real-world compressor behavior under varying external and setpoint-driven conditions, improving temperature control accuracy.

#### Fan power

Fan power depends on the outdoor temperature difference and follows a nonlinear correction function:

$$P_{\textrm{fan}} = P_{\textrm{fan, base}} \cdot \left( 0.5 + \left( 1 - \cos \left( \frac{\pi }{2} \cdot \frac{\Delta T_{\textrm{c}}}{K_f} \right) \right) \right)$$

where:

- $P_{\textrm{fan, base}}$: Baseline fan power;
- $K_f$: Fan power correction coefficient, determined by equipment characteristics and environmental factors.

#### Total power

The total power consumption of the air conditioning system is the sum of compressor power and fan power:

$$P_{\textrm{total}} = P_{\textrm{comp}} + P_{\textrm{fan}}$$

### Energy efficiency coefficient correction

The coefficient of performance (COP) of air conditioners varies with outdoor temperature conditions, impacting the system’s energy efficiency. It is corrected using a linear function based on the deviation from a reference temperature:

$$\text {COP} = \text {EER} - k_{\text {cop}} \cdot (T_{\text {out}} - T_{\text {ref}})$$

where:

- $\text {EER}$: Energy efficiency ratio under rated conditions;
- $k_{\text {cop}}$: Correction coefficient reflecting the decline in efficiency with increasing outdoor temperatures;
- $$T_{\text {ref}}$$: Reference temperature.

This dynamic COP adjustment ensures alignment with real-world performance, complementing the power model’s dependency on temperature differences.

### Dynamic cooling capacity adjustment

The cooling capacity dynamically responds to the indoor-outdoor temperature difference ($\Delta T_{\text {c}}$) and external environmental conditions, adjusted as follows:

$$\text {CoolingCapacity} = P_{\text {total}} \cdot \text {COP} \cdot k_{\text {cool}}$$

where:

- $P_{\text {total}}$: Total power consumption;
- $\text {COP}$: Coefficient of performance, corrected dynamically;
- $k_{\text {cool}}$: Cooling capacity adjustment factor, calculated as:
  $$k_{\text {cool}} = \left( 1 + K_{c1} \cdot \frac{\Delta T_{\text {c}}}{\Delta T_{\text {max}}} \right) \cdot \left( 1 - K_{c2} \cdot \frac{\Delta T_{\text {c}}}{T_{\text {ref}}} \right)$$
- $K_{c1}$: Indoor temperature difference adjustment coefficient;
- $K_{c2}$: External environment correction coefficient.

This formulation enhances the cooling capacity’s sensitivity to $\Delta T_{\text {c}}$, improving temperature regulation under varying thermal loads compared to the previous model.

### Dynamic temperature updates

The indoor temperature is updated dynamically at each time step based on the net heat balance:

$$T_{\text {in, new}} = T_{\text {in, old}} + \frac{Q - \text {CoolingCapacity}}{C_{\text {indoor}}}$$

where:

- $Q$: Total indoor heat load, including contributions from solar radiation, human activity, conductive transfer, and fresh air intake, corrected as:
  $$Q = Q_s + Q_c \cdot (1 - k_q \cdot S_{\text {fan}}) + Q_p + Q_{\text {fresh}} - Q_{\text {loss}}$$
- $k_q$: Heat load correction coefficient;
- $\text {CoolingCapacity}$: Dynamic cooling capacity, as defined above;
- $C_{\text {indoor}}$: Indoor thermal capacitance.

**Note:** For enclosed spaces typical of air conditioner usage, the heat transfer coefficient $k_a$ in the indoor heat load calculation is corrected using a scaling factor $c_a$, ensuring accurate load estimation. The updated process integrates $\Delta T_{\text {c}}$, $\Delta T_{\text {set}}$, power consumption, and COP, providing precise modeling under complex conditions.

## Simulation verification and result analysis

This study validates the effectiveness of the model through simulation. First, the temperature control and power output effects of a single air conditioner model are verified. Then, based on dynamic scenarios, the response of an air conditioner cluster is evaluated, and the impacts of environmental temperature, regional economic characteristics, and spatiotemporal changes on air conditioner energy consumption are analyzed. Finally, the effects of different temperature control strategies on the energy consumption of the air conditioner cluster are examined, further validating the model’s control response capability.

### Air conditioner cluster performance validation

This section validates the proposed model through simulation, focusing on air conditioner cluster performance. It evaluates averaged cluster metrics to confirm reliability, compares simulation methods to highlight the model’s strengths, and analyzes cluster responses to dynamic scenarios-spatiotemporal variations, temperature changes, and control strategies-demonstrating its robustness for intelligent load management.

**Fig. 8:**
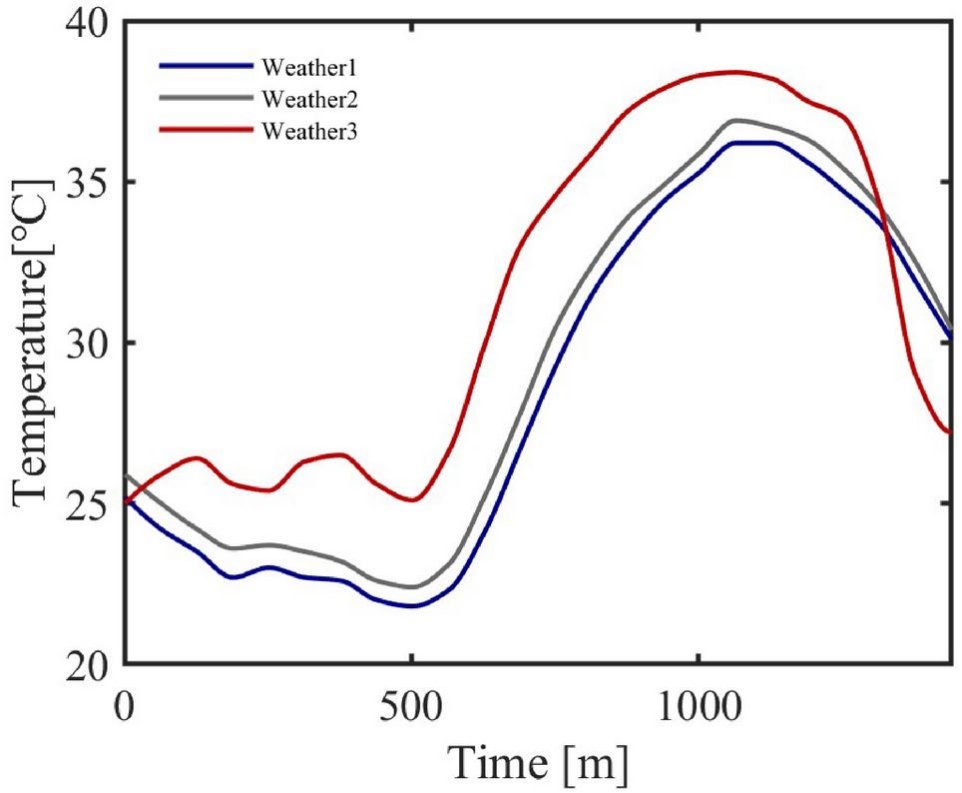
Micro Environment Temperature.

A 24-hour simulation period was set, with the date designated as a weekday. In residential areas, air conditioners were divided into bedroom and living room units, as shown in Table 4. The weather data were derived from real measurements in three adjacent districts of a northwestern city in China, as illustrated in Figure 8.

**Fig. 9:**
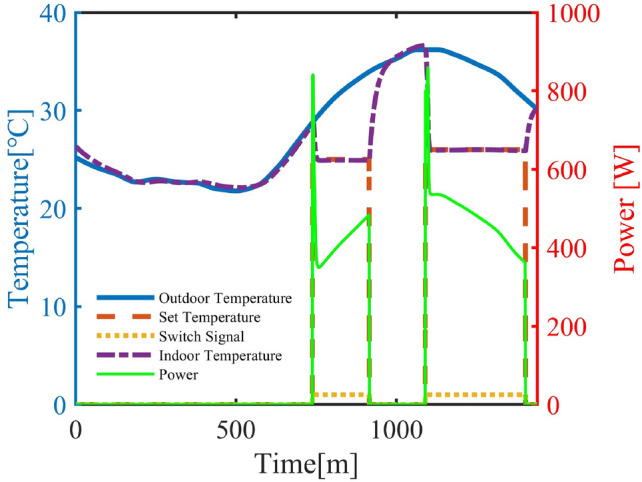
Single Air Conditioner Schematic Diagram.

**Table 4:** Regional Setup and Weather Conditions.

Region	Res(Bed/Live)	Adm	Ind	Com
Units	1174/779	716	540	252
Avg. Rated Power (W)	813/1168	1502	2090	1505
Weather	Weather1	Weather1	Weather3	Weather2

As an example, Figure 9 shows the simulation data curve of a single air conditioner in a residential area, illustrating the interrelationships among the modules. The left y-axis indicates temperature, while the right y-axis represents power output. The outdoor temperature is taken from “weather1” in Figure 8. The indoor temperature is composed of three parts: its initial value is randomly generated from the outdoor temperature; when the air conditioner is off, the temperature is calculated using the indoor temperature model; and when the air conditioner is on, the temperature is jointly determined by the indoor temperature and the power model. The setpoint temperature data comprehensively considers outdoor temperature, resident occupancy status, temperature thresholds, and tolerance time, which is normalized to obtain on/off states. The power output is derived from the power model. This schematic indicates that the air conditioner responds rapidly to the on/off signal, can stably track the setpoint temperature, and that power fluctuations reflect the instantaneous peak during start-up as well as efficiency reduction effects when the temperature difference is large.

**Table 5:** Performance Statistics Across Representative Regions.

Area	Res (Bed/Live)	Adm	Ind	Com
Stabilization Time (min)	11	5	5	6
Steady-State MAE (°C)	0.46	0.26	0.29	0.27
Power-$\Delta T_c$ Corr. ($\rho$)	0.8398	0.7961	0.7564	0.8055
Avg. On-Time (min)	218/504	316	727	716
Avg. Energy (kWh)	1.67/4.93	3.66	12.57	8.86
Peak Load Periods	00:09-05:27/18:05–24:00	11:32-17:21	10:14–18:55	12:19–22:59

Table 5 compares the air conditioner cluster performance indicators across four regions. Stabilization time is defined as the duration required after an on-signal until $|\Delta T_{\text {set}}| < 1.5^\circ \textrm{C}$. Because residential areas have relatively lower rated power, stabilization requires approximately 11 minutes, whereas the functional areas stabilize within 5–6 minutes. The steady-state mean absolute error (MAE), calculated by

$$MAE = \frac{1}{n} \sum _{i=1}^{n} \bigl |y_i - \hat{y}_i\bigr |$$

ranges from 0.26 to 0.46°C, reflecting stable tracking of the setpoint temperature.

The Pearson correlation coefficient between the indoor-outdoor temperature difference and power is given by

$$\rho = \frac{\sum (P_i - \overline{P}) (\Delta T_{c,i} - \overline{\Delta T_c})}{\sqrt{\sum (P_i - \overline{P})^2 \sum (\Delta T_{c,i} - \overline{\Delta T_c})^2}}$$

with values in the 0.75–0.85 range, indicating a moderately strong positive correlation. The average on-time, average energy consumption, and peak load periods reflect the operational and economic characteristics of the air conditioner clusters in each region. Overall, these indicators collectively illustrate the reliability of the model for cluster load management.

### Simulation method comparison and analysis

To address the limitations of real-world data, this study employs simulated air conditioner operation data to validate the effectiveness of the proposed approach. Centered on user activity states, the behavior-based model generates $T_{\text {set}}$ and $S_{\text {fan}}$ by incorporating ambient temperature, threshold parameters, and tolerance time. Using the administrative district case (716 air conditioners, 1440 minutes), we compare three common methods: the randomized switching model and the threshold-triggered model alongside the proposed method. The only differences lie in how setpoint temperature and on/off signals are generated, highlighting the advantages of behavioral modeling for intelligent load management.

#### Method description

**1. Proposed Behavior-Based Model** This method dynamically generates $T_{\text {set}}$ and $S_{\text {fan}}$ based on user activity states (e.g., the typical “9-to-5” routine in an administrative district), $T_{\text {out}}$, threshold temperature, and tolerance time.

**2. Randomized Switching Model** The on/off signal $S_{\text {fan}}$ is generated via segment-based probabilities, dividing the day into three time periods:

$$S_{\text {fan}} \sim \text {Bernoulli}\bigl (P_{\text {on}}(t)\bigr ), \quad P_{\text {on}}(t) = {\left\{ \begin{array}{ll} 0.2, & t \in [1, 540]\cup [1021, 1440],\\ 0.8, & t \in [541, 1020]. \end{array}\right. }$$

where:

- $P_{\text {on}}(t)$: Probability that the air conditioner is switched on at time $t$.Once turned on, it remains active for 120–300 minutes.

**3. Threshold-Triggered Model** The on/off signal $S_{\text {fan}}$ is determined by comparing indoor temperature $T_{\text {in}}$ with preset thresholds:

$$S_{\text {fan(t)}} = {\left\{ \begin{array}{ll} 1, & T_{\text {in}}(t)> T_{\text {upper}} = 28^\circ \textrm{C},\\ 0, & T_{\text {in}}(t) < T_{\text {lower}} = 24^\circ \textrm{C},\\ S_{\text {fan(t-1)}}, & T_{\text {lower}} \le T_{\text {in}}(t) \le T_{\text {upper}}. \end{array}\right. }$$

When switched on, $T_{\text {set}}$ is uniformly sampled from 20–26°C and remains constant during the on period.

#### Simulation results and analysis

**Fig. 10:**
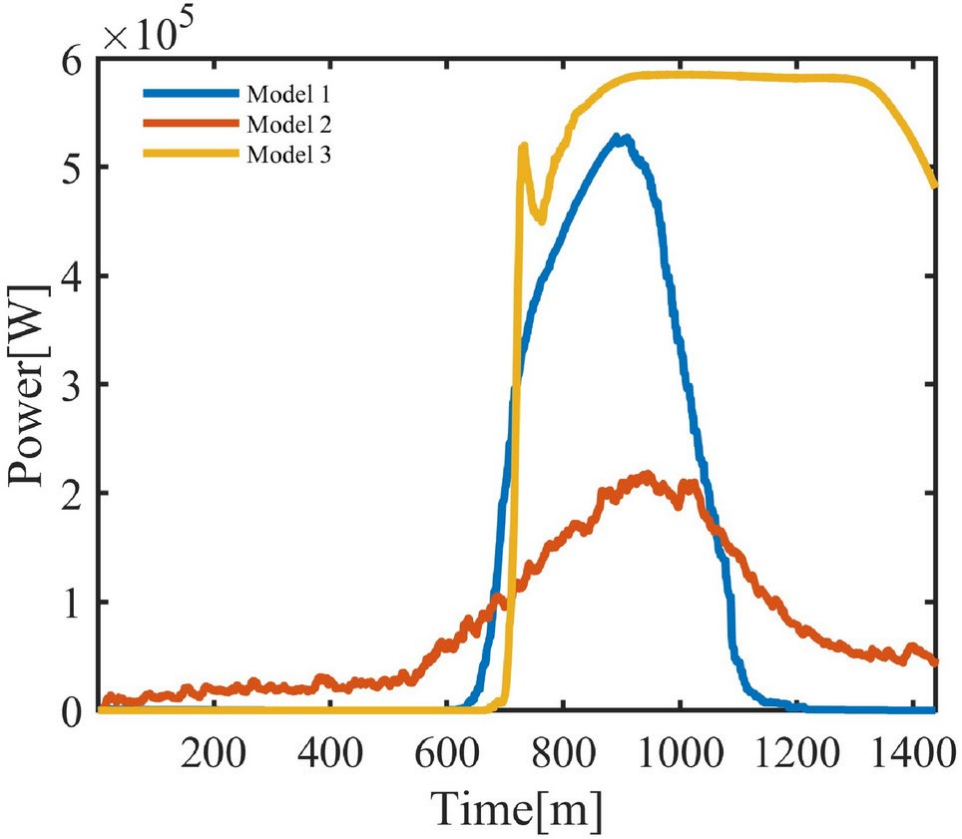
Administrative District Power Under Three Models.

Figure 10 shows the average power curves of the three methods. The power of the behavior-based model concentrates between 10:00 and 20:00, reflecting the routine of an administrative district. In the randomized switching model, power distribution is scattered, with lower peak and total power, and does not account for $T_{\text {out}}$. In the threshold-triggered model, operation persists under high outdoor temperatures, maintaining high power output until $T_{\text {out}}$ decreases.

**Table 6:** Performance Indicators of the Three Models.

Indicator	Behavior-Based	Randomized Switching	Threshold-Triggered
Stabilization Time (min, $\pm 1.5$)	5	9	9
Steady-State MAE (°C)	0.26	0.41	0.29
Avg. On-Time (min)	316	268	709
Avg. Energy (kWh)	3.66	3.13	9.45
Peak Usage Period	11:32-17:21	11:34-17:29	12:27-24:00
Power-$\Delta T$ Corr. ($\rho$)	0.7961	0.6618	0.7734

As shown in Table 6, the behavior-based model achieves the shortest stabilization time (5 minutes) and lowest MAE (0.26°C), indicating accurate tracking. In contrast, the randomized switching model requires 9 minutes for stabilization (+80%), with its MAE increasing to 0.41°C (+57.7%). Although on-time and energy usage are reduced by about 15%, its response is relatively insufficient. The threshold-triggered model, on the other hand, runs 709 minutes on average, consumes 9.45 kWh-more than double-and extends its peak period until midnight, resulting in lower efficiency. Overall, the behavior-based model more accurately reflects user activity states and regional characteristics, enhancing its applicability to intelligent load management.

### Cluster response in dynamic scenarios

#### Cluster response to temporal-spatial changes

**Cluster Response to Date Types**

**Fig. 11:**
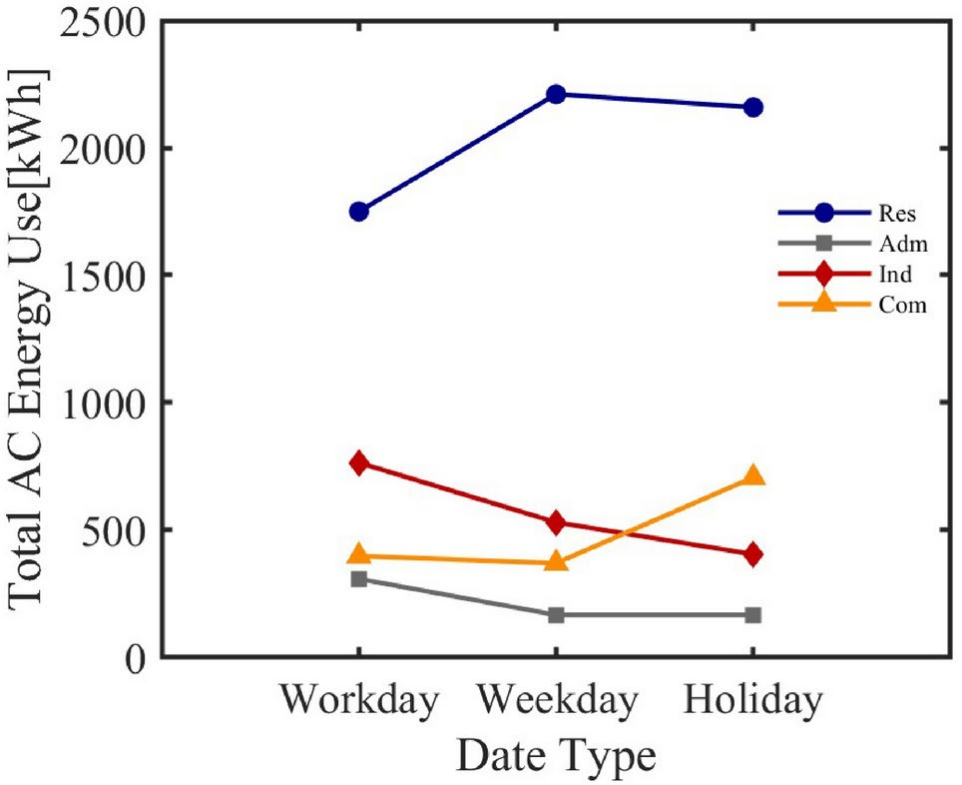
Cluster Energy Date Types.

The model considers the energy consumption variation of air conditioner clusters on different types of dates (working days, weekends, holidays). The analysis results shown in Figure 11 show that the energy consumption of air conditioners in residential areas increases on weekends and holidays, which aligns with the change in residential activity status. In contrast, energy consumption in industrial and administrative areas decreases on weekends and holidays, reflecting the service nature of these areas.

**Cluster Response to Spatial Distance**

Different commuting times reflect the spatial influence on personnel movement in different regions. In the model, the personnel dynamics of administrative and industrial areas are relatively stable, while commuting time mainly affects the air conditioner cluster response in commercial areas. The following shows the progressive changes of three commuting time schemes and the resulting changes in air conditioner power-on quantity and energy consumption in the commercial area.Three commuting time schemes are shown in Table 7, 8 and 9.

**Table 7:** Commuting Time in Commercial Areas (5–15 minutes).

Area	adm	ind	com1	com2
area1	15–25	40–50	5–10	10–15
area2	10–20	40–50	10–15	5–10
area3	10–20	20–30	10–15	10–15

**Table 8:** Commuting Time in Commercial Areas (55–75 minutes).

Area	adm	ind	com1	com2
area1	15–25	40–50	55–60	60–75
area2	10–20	40–50	60–75	55–60
area3	10–20	20–30	60–75	60–75

**Table 9:** Commuting Time in Commercial Areas (110–160 minutes).

Area	adm	ind	com1	com2
area1	15–25	40–50	110–130	140–160
area2	10–20	40–50	140–160	110–130
area3	10–20	20–30	140–160	140–160

**Fig. 12:**
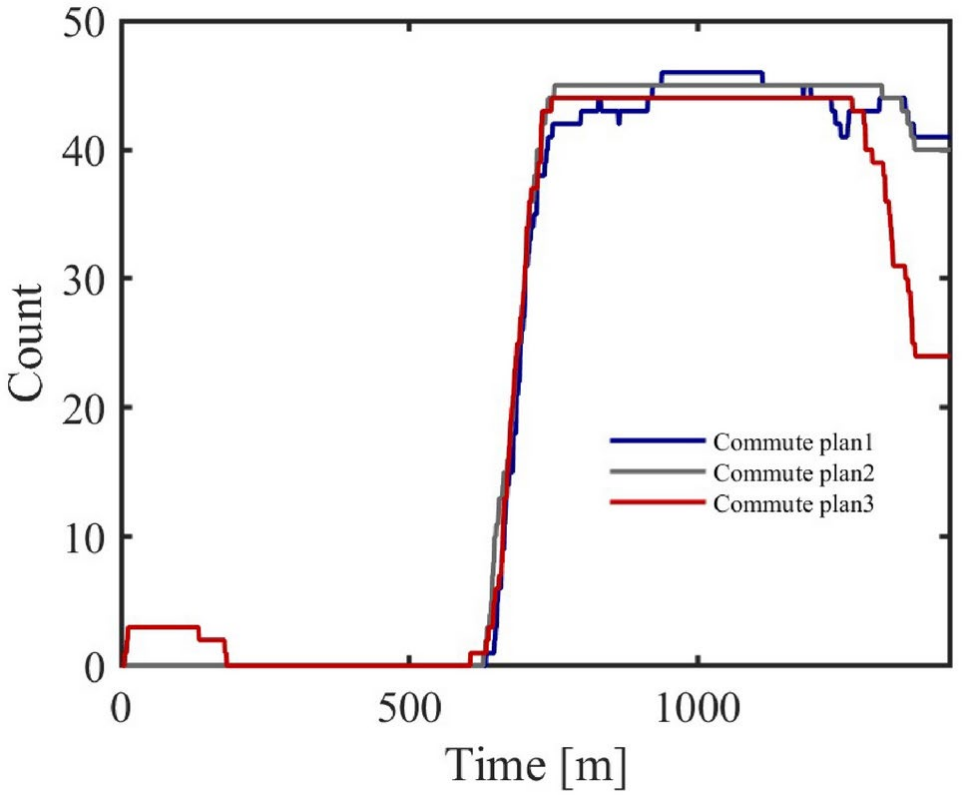
Air Conditioner Power-On Quantity under Different Commuting Schemes.

Figure 12 shows the variation in air conditioner power-on quantity under different commuting schemes. As commuting time increases, the response of commercial area air conditioners becomes more noticeable. Especially in Scheme 3, with the increase in commuting time, the power-on quantity in the commercial area shows a significant decrease during off-hours.

**Fig. 13:**
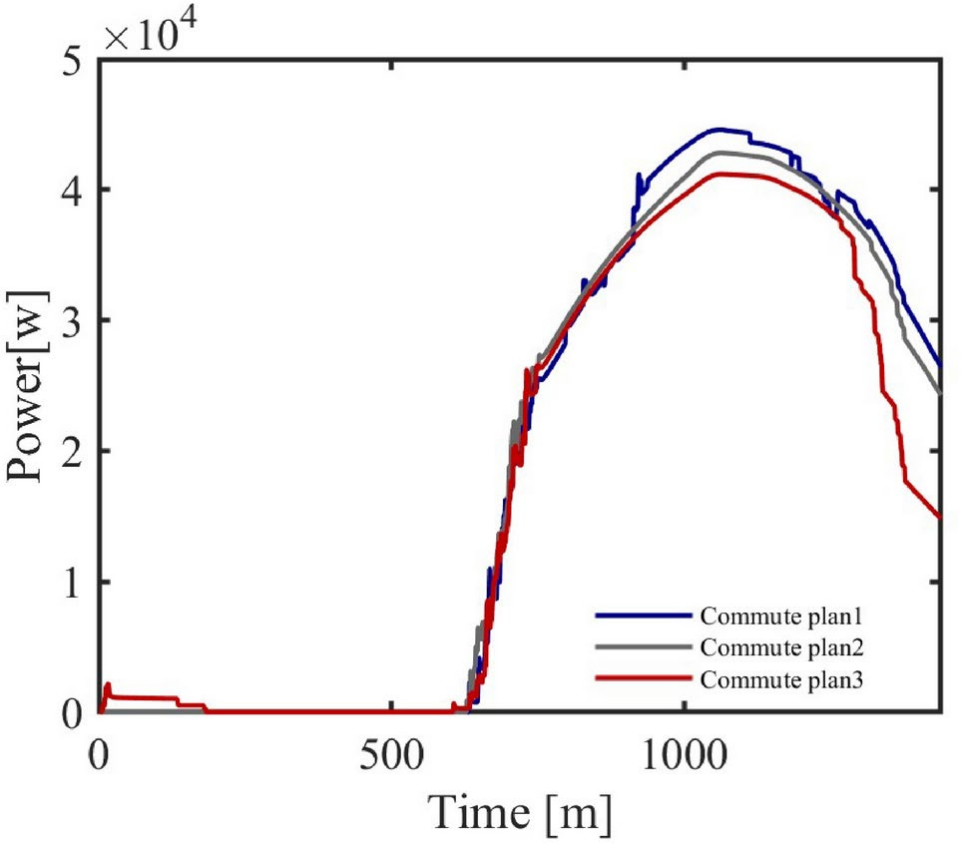
Energy Consumption Change with Commuting Time.

Figure 13 shows the relationship between increased commuting time and energy consumption in commercial areas. As the commuting distance between residential and commercial areas increases, energy consumption in the commercial area gradually decreases, with the energy consumption drop occurring earlier. This indicates that commuting time has a noticeable impact on the energy consumption of the commercial area air conditioner cluster.

#### Cluster response to environmental temperature

Temperature, as an important environmental factor for air conditioner operation, is a key indicator for verifying the dynamic performance of the air conditioner cluster. By setting a ${2}^{\circ }$C temperature gradient, the response characteristics of the air conditioner cluster in terms of average power consumption, number of units switched on, and daily power consumption are observed at different temperatures.

**Fig. 14:**
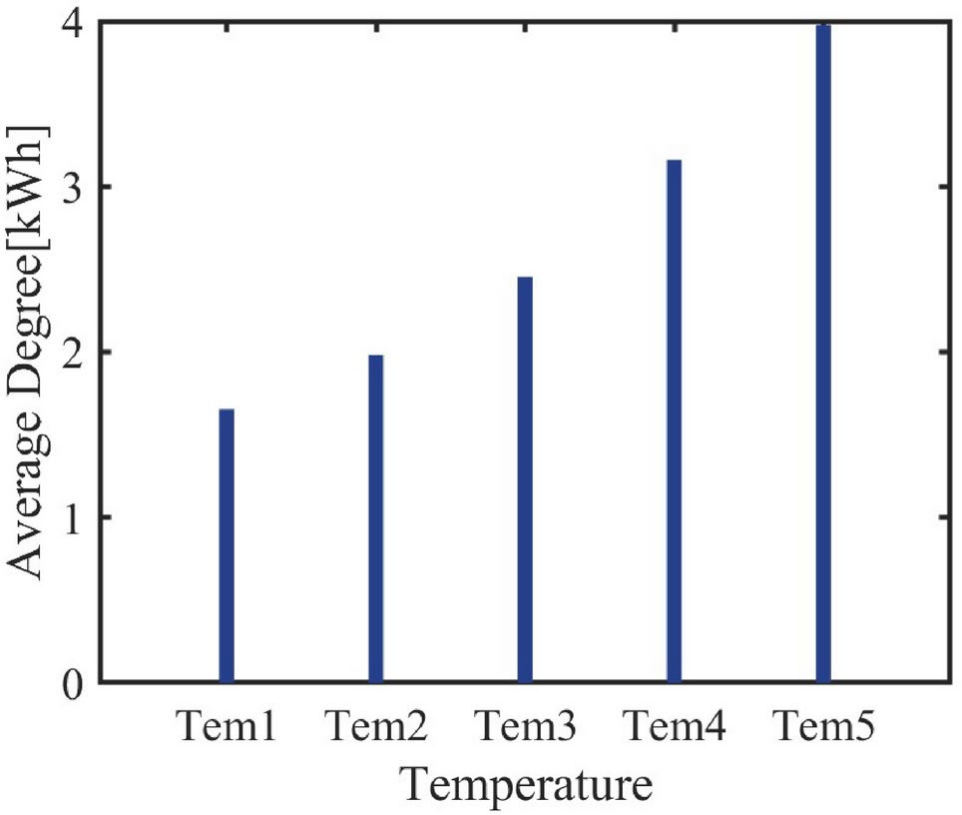
Average Energy Consumption Bar Chart.

Figure 14 shows that, as the temperature increases, the daily average energy consumption of the air conditioner cluster increases in a progressive manner with each ${2}^{\circ }$C rise. Specifically, with every ${2}^{\circ }$C increase, the daily average energy consumption of the cluster rises by 20%, 23.7%, 29%, and 26% respectively, while the number of air conditioners turned on increases by 6%, 10.4%, 35.9%, and 42.8%.

**Fig. 15:**
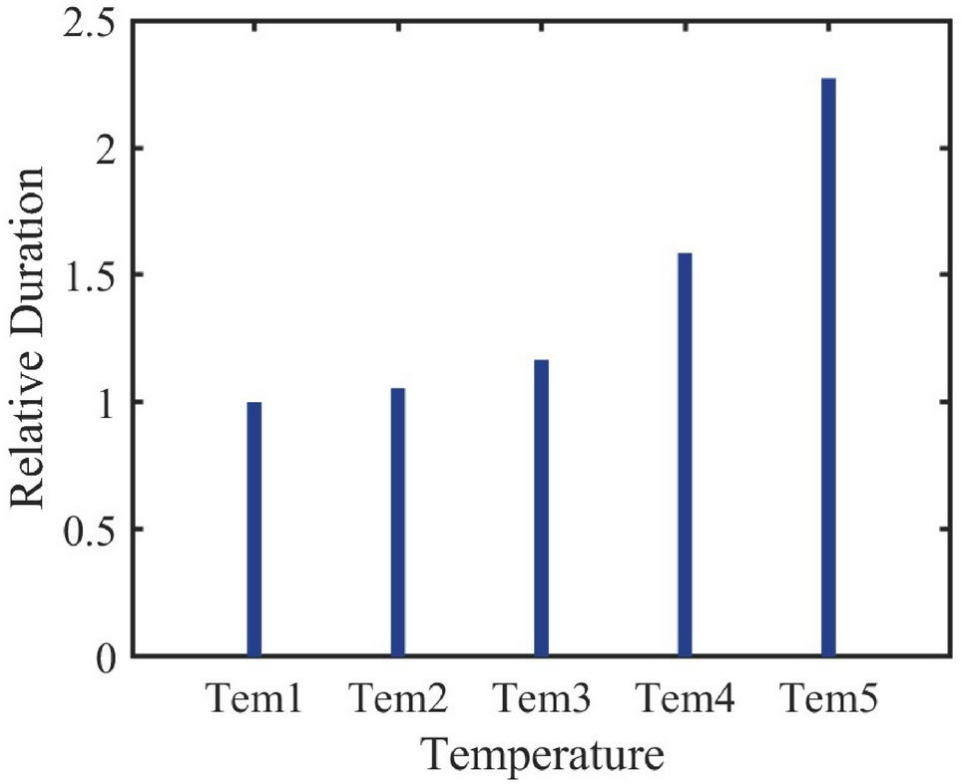
Number of Air Conditioners Turned On Bar Chart.

Figure 15 further confirms the impact of temperature on the response of the air conditioner cluster.

**Fig. 16:**
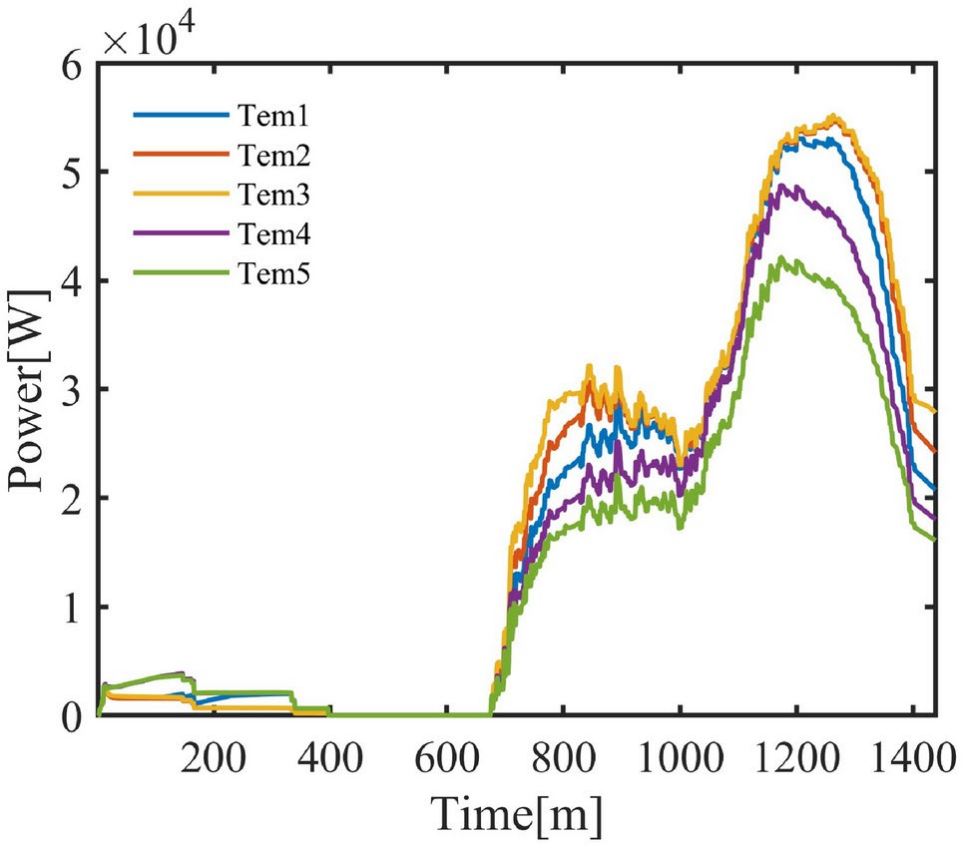
Daily Power Curve of the Air Conditioner Cluster at Different Temperatures.

From Figure 16, it is evident that the incremental increase in air conditioning power consumption varies at different temperatures and times. For residential areas, the increase in power consumption during the night becomes noticeable when the outdoor temperature rises by ${6-8}^{\circ }$C, indicating that lower temperatures have less impact on air conditioning energy use. In contrast, at higher temperatures, the increase in air conditioning usage becomes more pronounced, which is consistent with common observations of air conditioner usage.

### Cluster response to set temperature regulation

Set temperature, a key parameter in air conditioner cluster regulation, directly affects energy consumption. This study examines its impact through simulation, incrementally reducing set temperature by 1 °C across five ranges. Control strategies were applied to residential and industrial regions, with a random subset of units regulated. Energy consumption increments were analyzed based on set temperature changes and the proportion of controlled units.

**Fig. 17:**
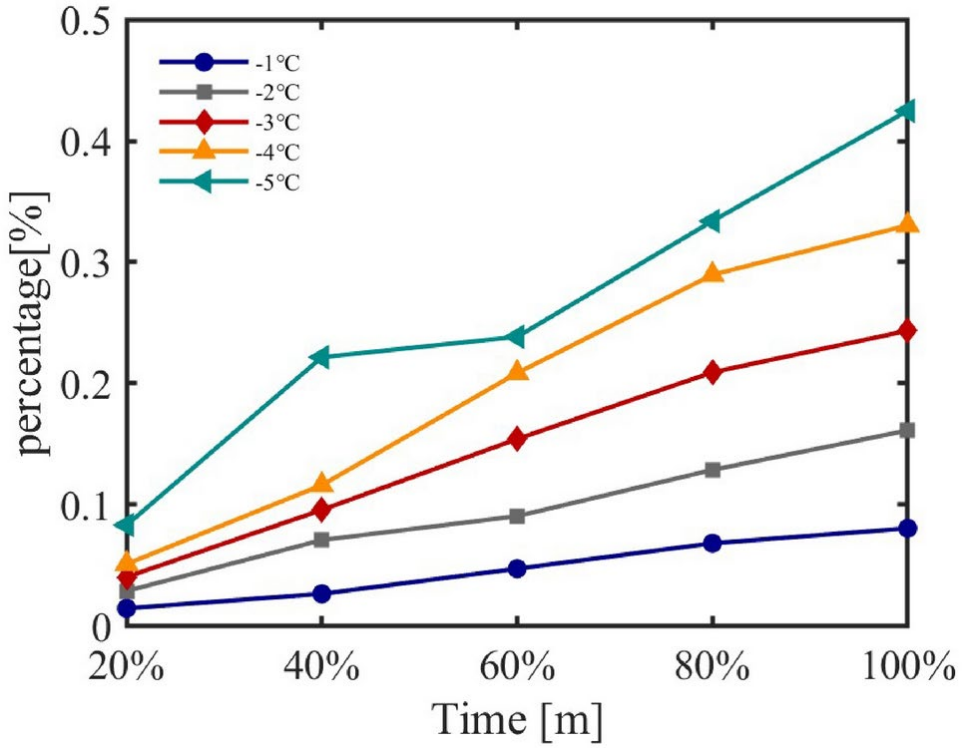
Energy Consumption Increments under Different Set Temperatures for Residential Areas.

**Fig. 18:**
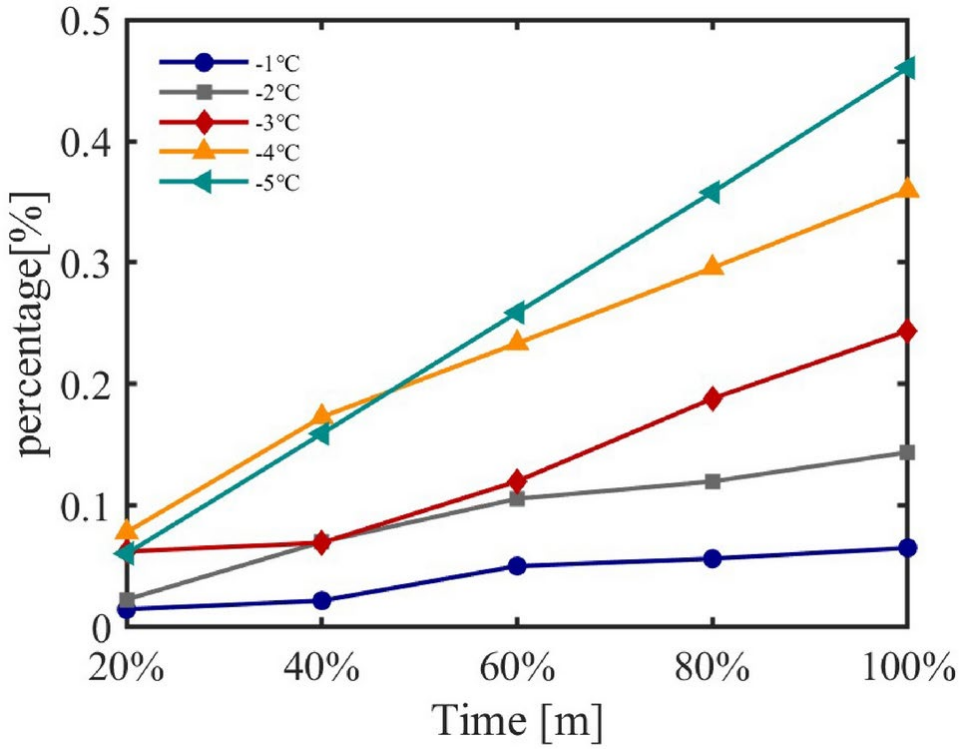
Energy Consumption Increments under Different Set Temperatures for Industrial Areas.

Figure 17 shows energy consumption increments in residential clusters, revealing a rising trend as set temperature decreases, with fluctuations attributed to varying unit proportions and specifications. Similarly, Figure 18 illustrates increments in industrial clusters, displaying notable variability under regulation.

Trends across regions indicate energy consumption increases with decreasing set temperature, though not linearly, due to fluctuations from user mobility and unit power variations. Default proportions of air conditioners are 77.7% (residential) and 7.0% (industrial), with interquartile power ranges of 460 W and 1325 W, and standard deviations of 365 W and 830 W, respectively. At 100% regulation, a 1 °C reduction consistently raises energy use by approximately 8% daily.

The model effectively captures load dynamics under varying set temperatures and ambient conditions, aligning with findings that energy use reflects temperature, user preferences, and habits^41,42^. These results validate the model’s accuracy and its utility for intelligent load management in distribution networks.

## Conclusions and future prospects

This study develops an air conditioning cluster response model for intelligent load management in distribution networks. By integrating heat balance and dynamic temperature updates, the model accurately simulates cluster switching states and energy consumption across diverse regions. Simulations reveal that energy use varies with ambient temperature, regional economic traits, activity patterns, and set temperature adjustments, significantly affecting cluster load dynamics.

Multidimensional simulations-covering environmental, economic, and spatiotemporal factors-validate the model’s ability to reflect real-world cluster behavior and support efficient control strategies for energy savings. Set temperature adjustments further demonstrate its potential for precise load management under dynamic conditions, providing robust decision support for distribution networks.

Future improvements include:

Refined Behavioral Modeling: Incorporate advanced activity patterns for greater precision. Multi-Device Integration: Extend to appliances like water heaters and electric vehicles. Data-Driven Enhancement: Use real-world data and machine learning to optimize parameters. Smart Control Algorithms: Develop real-time, optimized load management strategies. Regional Coordination: Explore cross-regional cluster control for efficient energy scheduling. In conclusion, ongoing refinements will enhance the model’s role in smart grid forecasting, energy optimization, and decision-making.

## Data Availability

The datasets generated and/or analyzed during the current study are available from the first author (ZhiYong Zhang, email: 962086671@qq.com) on reasonable request.

## Appendix A: Model parameter values

See Table 10.

**Table 10:** Model Parameter Values.

Parameter	Symbol	Value
Solar irradiation coefficient	$k_s$	0.008
Heat transfer coefficient	$k_a$	2.5
Heat loss coefficient	$k_{loss}$	0.35
Heat capacity coefficient	$k_c$	6
Indoor human heat coefficient	$k_p$	1.5
Efficiency degradation coefficient	$k_{cop}$	0.025
Reference temperature	$T_{\text {ref}}$	25
Maximum temperature difference	$\Delta T_{\text {max}}$	15
Fan power correction coefficient	$K_f$	16
Cooling capacity adjustment coefficient (indoor)	$K_{c1}$	5.89
Cooling capacity adjustment coefficient (external)	$K_{c2}$	0.14
Heat transfer correction coefficient	$c_a$	0.05
Fresh air mass flow rate	$\dot{m}_{\text {fresh}}$	0.005 kg/s
Specific heat capacity of air	$c_p$	1.005 $\hbox {kJ}/\hbox {kg}\cdot \hbox {K}$
Compressor base power coefficient	$a$	0.75
Compressor scaling coefficient	$b$	0.02
Control factor coefficient 1	$C_{f1}$	2.04
Control factor coefficient 2	$C_{f2}$	4.03
Heat load correction coefficient	$k_q$	0.65
